# High-resolution DNA methylome reveals that demethylation enhances adaptability to continuous cropping comprehensive stress in soybean

**DOI:** 10.1186/s12870-019-1670-9

**Published:** 2019-02-18

**Authors:** Xilong Liang, Xue Hou, Jianying Li, Yiqiang Han, Yuxian Zhang, Naijie Feng, Jidao Du, Wenhui Zhang, Dianfeng Zheng, Shumei Fang

**Affiliations:** 1 Heilongjiang Bayi Agricultural University, Daqing, 163319 China; 2 Daqing Branch of Heilongjiang Academy of Agriculture Science, Daqing, 163316 China

**Keywords:** Continuous cropping comprehensive stress, DNA demethylation, Demethylase, Differentially methylated genes, Metabolism analysis, Soybean

## Abstract

**Background:**

Continuous cropping stress involves such factors as biological barriers, allelopathic autotoxicity, deterioration of soil physicochemical properties, and soil fertility imbalance and is regarded as a kind of comprehensive stress limiting soybean yield and quality. Genomic DNA methylation is an important regulatory mechanism for plants to resist various environmental stresses. Therefore, it is especially worthwhile to reveal genomic methylation characteristics under stress and clarify the relationship between DNA methylation status and continuous cropping stress adaptability in soybean.

**Results:**

We generated a genome-wide map of cytosine methylation induced by this kind of comprehensive stress in a tolerant soybean variety (Kang Xian 2, KX2) and a sensitive variety (He Feng, HF55) using whole-genome bisulfite sequencing (WGBS) technology. The expression of DNA demethylase genes was detected using real-time quantitative PCR (qRT-PCR). The functions of differentially methylated genes (DMGs) involved in stress response in biochemical metabolism and genetic information transmission were further assessed based on Gene Ontology (GO) annotation and Kyoto Encyclopedia of Genes and Genomes (KEGG) pathway analysis. The results showed that genomic DNA demethylation was closely related to continuous cropping comprehensive stress adaptability in soybean, which was further verified by the increasing expression of DNA demethylases ROS1 and DML. The demethylation of mCpG and mCpHpG (mCpApG preferred) contexts was more critical, which mainly occurred in gene-regulatory regions at the whole-chromosome scale. Moreover, this kind of stress adaptability may be related to various stress responders generated through strengthened glucose catabolism and amino acid and fatty acid anabolism, as well as fidelity transmission of genetic information.

**Conclusions:**

Genomic DNA demethylation was closely associated with continuous cropping comprehensive stress adaptability, highlighting the promising potential of screening continuous cropping-tolerant cultivars by DNA methylation index and further exploring the application of DNA demethylases in soybean breeding.

**Electronic supplementary material:**

The online version of this article (10.1186/s12870-019-1670-9) contains supplementary material, which is available to authorized users.

## Background

Soybean (*Glycine max* [L.] Merr.), an agricultural product used for grain, cooking oil, fodder, and important industrial raw materials, is a continuous global staple crop [1, 2]. Soybean plants are also important for soil fertility because they can fix atmospheric nitrogen through symbiosis with microbes in the rhizosphere [3]. However, due to salinization, desertification, the growing population, and other reasons, the area of arable land has decreased considerably over the last few decades [4, 5]. The increasing demand for soy products and reduced cultivated land acreage have resulted in large areas of soybean coming under continuous cropping stress, especially in China [6–8]. For instance, the acreage devoted to soybean cultivation under continuous cropping accounted for more than 40% of the whole soybean planting area in 2012 in Heilongjiang Province, Northeast China [9]. After long-term continuous cropping, the crop may have poor growth due to continuous cropping obstacles including biological barriers, allelopathic autotoxicity of plants, the deterioration of soil physicochemical properties, and soil fertility imbalance, leading to low yields and poor quality [10–13]. Therefore, the obstacle of continuous cropping, a kind of comprehensive adversity, has been one of the bottlenecks restricting soybean yield increases and quality improvement.

When crops are exposed to stressful conditions, they will resort to various strategies to minimize the effects of stress, such as tolerance, resistance and avoidance. These strategies usually arise from changes in related gene expression [14, 15]. DNA methylation is an indispensable epigenetic mechanism for normal plant development under adverse conditions that can result in stable alterations in gene expression without changes in the underlying DNA sequence [16–19].

In plants, DNA methylation commonly occurs at cytosine sites (where a methyl group is added at the 5′ position to form 5-methylcytosine) in either symmetrical CpG and CpHpG sequence contexts or asymmetrical CpHpH (where H is A, C, or T) contexts [20, 21]. Cytosine methylation in all sequence contexts is established through the RNA-directed DNA methylation (RdDM) pathway guided by 24-nt small interfering RNAs (siRNAs), in which the DNA methyltransferase Domains Rearranged Methyltransferase 2 (DRM2) is recruited to mediate de novo methylation. CpG and CpHpG methylations are maintained during subsequent rounds of DNA replication because of their symmetrical nature by DNA methyltransferase 1 (MET1) and chromomethylase 3 (CMT3), respectively, whereas asymmetrical CpHpH methylation is maintained by the RdDM pathway and chromomethylase 2 (CMT2) [22]. DNA methylation can also be removed by either passive DNA demethylation (failure to maintain methylation after replication) or active DNA demethylation (active removal by some enzymes). In plants, active DNA demethylation is mediated by members of the bifunctional DNA glycosidase subfamily, including Demeter (DME), Repressor of Silencing 1 (ROS1) and Demeter-like (DML). These enzymes can not only catalyse the hydrolysis of a glycosylic bond between the methylcytosine base and deoxyribose but also cleave the DNA backbone at abasic sites to form a single-nucleotide gap that will be filled with an unmethylated cytosine nucleotide by polymerase and ligase [21, 23–28].

By these DNA methylation or demethylation processes, DNA methylation can be dynamically regulated and maintained at a proper level in plants. When crop plants encounter environmental stresses, genomic DNA methylation levels will be changed to adapt to the challenge [29]. Diverse environmental stresses, such as cold, aluminium, herbicide, salt, drought stress, and nutrient stress, can induce heritable alteration in DNA methylation in plants [16, 30–34]. For example, cold stress causes strong genome-wide DNA demethylation in maize seedlings, and the transcription of some demethylated genes increases in response to cold [29, 35]. Salinity stress also induces DNA demethylation events in a tolerant *Setaria italica* L. cultivar, and the expression of stress-responsive genes is modulated [36]. However, in *Medicago truncatula*, salinity stress increases DNA methylation level as a stress-adaptive response [30]. Consequently, it is reasonable for us to assume that methylation alteration might be important for soybean plants to adapt to the comprehensive stress of continuous cropping. Up to now, there has been no report discussing the relationship between soybean continuous cropping adaptation and genomic DNA methylation.

In this study, we generated DNA methylomic maps of soybean varieties sensitive (He Feng 55, HF55) and tolerant (Kang Xian 2, KX2) to continuous cropping and examined their methylation changes induced by continuous cropping comprehensive stress using whole-genome bisulfite sequencing (WGBS) technology, which can accurately quantify whole-genome methylation at single-base resolution. The methylation levels of total C, CpG, CpHpG, and CpHpH contexts; their distribution characteristics in the genome, on every chromosome and in specific gene regions; and their changes under continuous cropping comprehensive stress were revealed. Based on demethylation obtained in this work, the expression levels of DNA demethylase genes, including *ROS1*, *DME* and *DML*, were detected by real-time quantitative PCR (qRT-PCR). Moreover, analysis of Gene Ontology (GO) annotation and Kyoto Encyclopedia of Genes and Genomes (KEGG) pathways of differentially methylated genes (DMGs) were performed to identify some genes involved in metabolic processes and fidelity transmission of genetic information associated with response to continuous cropping comprehensive stress. This work made it clear that genomic DNA demethylation was closely associated with continuous cropping comprehensive stress adaptability, reinforcing the understanding of DNA demethylation knowledge applied to continuous cropping-tolerant cultivars breeding in soybean. To the best of our knowledge, this report is the first detailing the soybean genomic DNA methylation characteristics induced by continuous cropping comprehensive stress, corresponding demethylase activity, and possible metabolic mechanism and gene regulation caused by demethylation related to this kind of comprehensive stress adaptation.

## Results

### Bisulfite sequencing reveals that demethylation is closely related to continuous cropping resistance in soybean

**Fig. 1:**
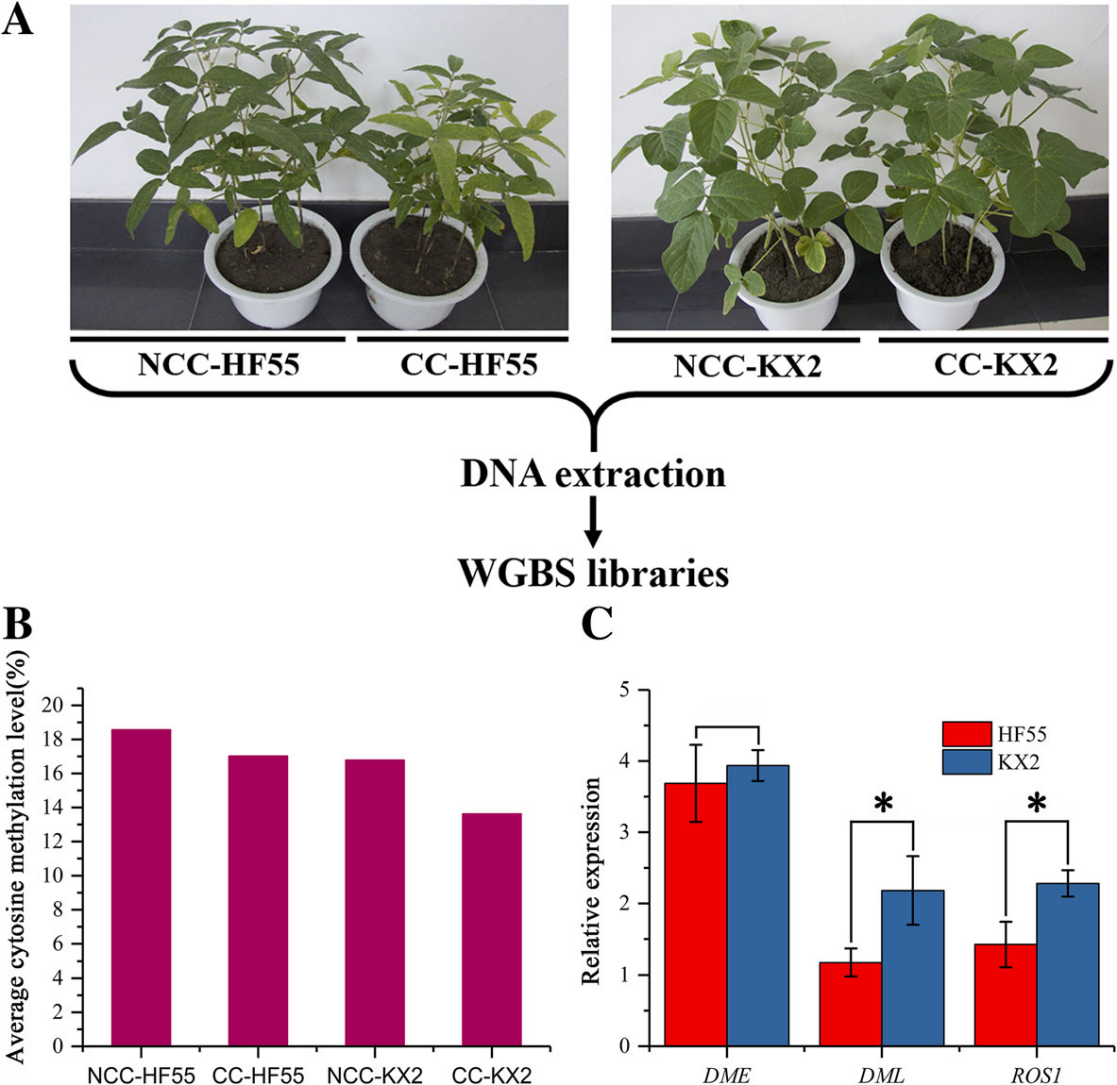
DNA methylomes of continuous cropping sensitive and tolerant soybean varieties and expression analysis of some demethylase genes. **a** Soybean plant morphology of sensitive HF55 and tolerant KX2 under continuous cropping stress. The tolerant variety was verifiable by its phenotype 60 days after sowing. **b** Whole-genome methylation levels (mC/C ratios) in both varieties under continuous cropping comprehensive stress and the non-continuous cropping condition. **c** Expression analysis of the demethylase genes *DME*, *DML* and *ROS1* under continuous cropping stress. (NCC: non-continuous cropping; CC: continuous cropping; HF55: He Feng 55; KX2: Kang Xian 2; same as below)

Before analysing genomic methylation, we verified the different adaptabilities of sensitive HF55 and tolerant KX2 by investigating some morphological indexes including plant height, leaf area, stem and leaf dry weight, nodule number, nodule dry weight and chlorophyll content under continuous cropping comprehensive stress (Fig. 1a, Additional file 1: Table S1). On the basis of those data, bisulfite-seq analysis was performed to obtain base-pair-resolution DNA methylomes using an Illumina HiSeq 2000 and the WGBS method. The WGBS library is the most comprehensive, highest-resolution method for detecting cytosine methylation (5mC) to reveal DNA methylation patterns and variation on a genome-wide scale, especially in the CpHpG and CpHpH contexts [37, 38]. Cytosine methylation of non-CpG sites is extensive (more than 30% of the total 5mC) in plant genomes [39, 40]. Two cultivars were used to construct the WGBS libraries under continuous and non-continuous cropping conditions, and 33 Gb of sequence data per sample was generated, which covered more than 80% of sequences of each chromosome and gene region (Additional file 1: Figure S1, Table S2, Table S3).

In this work, the whole-genome methylation levels (the ratio of methylcytosine to cytosine) of each sample were first calculated. The results showed that the methylation ratio of tolerant KX2 and sensitive HF55 was 16.78 and 18.57% under non-continuous cropping conditions, respectively. In contrast, both cultivars displayed demethylation when they were exposed to continuous cropping stress. The degree of demethylation in KX2 was 18.77%, which was higher than that in HF55 (8.35%) (Fig. 1b). These results indicate that the tolerant soybean variety KX2 had a higher ability than the sensitive variety HF55 to adjust its DNA methylation levels upon exposure to continuous cropping stress, suggesting a link between the plasticity of DNA methylation and plant performance under continuous cropping stress.

To confirm the reliability of the above results, we further detected the expression of some important DNA demethylase genes, including *DME*, *DML* and *ROS1*, by qRT-PCR. The results revealed that these genes were all up-regulated in both KX2 and HF55 under continuous cropping conditions (Fig. 1c). In particular, compared to the *DML* and *ROS1* gene expression levels in HF55, those in KX2 were increased by 85.9 and 60.1%, respectively (both *P* < 0.05). Therefore, genomic DNA demethylation was enhanced by increasing *DML* and *ROS1* expression, further indicating that demethylation was closely related to continuous cropping resistance of soybean.

### Demethylation of mCpG and mCpHpG contexts is more important than that of the mCpHpH context for resisting continuous cropping comprehensive stress in soybean

**Fig. 2:**
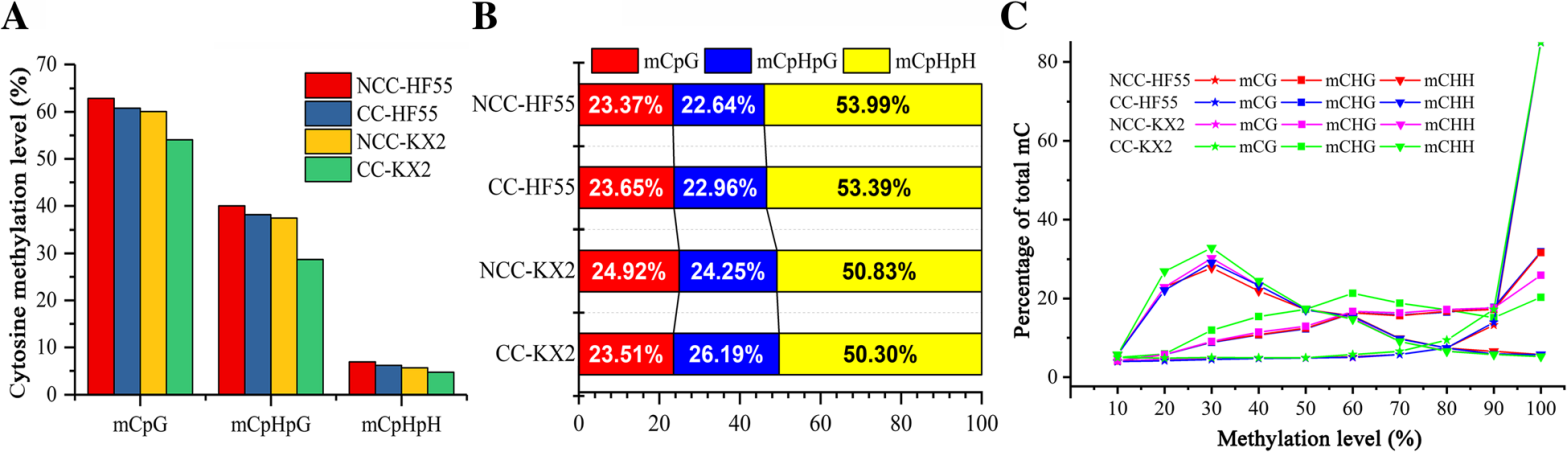
DNA methylation status of each sequence context in two soybean cultivars under different conditions. **a** Percentage of mCs at CpG, CpHpG, and CpHpH sites (H = C, T, or A). **b** The relative fraction of mCs identified for each sequence context (CpG, CpHpG and CpHpH). **c** Distribution of mC methylation level in each sequence context. Only mCs covered by at least 5 reads were used to calculate methylation level

The total DNA methylation levels and characteristics were further estimated at CpG and non-CpG (CpHpG and CpHpH) sites. The level of mCs in CpG dinucleotides was 62.86 and 60.08% in HF55 and KX2, respectively, which was higher than that of CpHpG sequences (40.02% in HF55 and 37.41% in KX2). CpHpH contexts only had a low methylation rate (6.92% in HF55 and 5.65% in KX2) (Fig. 2a). However, interestingly, the percentages of total methylcytosine events that occurred in the three sequence contexts were not consistent with the DNA methylation levels. Among the contexts, mCpHpH accounted for the highest proportion (53.99 and 50.83% in HF55 and KX2, respectively) (Fig. 2b), while the proportion for mCpG was only 23.37 and 24.92% in HF55 and KX2, respectively, similar to that in CpHpG (22.64 and 24.45% in HF55 and KX2). These contrasting results indicated that the methylation ratio in the CpG context was highest in the soybean genome, but in terms of the number of mCs, the mCpG and mCpHpG contexts had much lower rates than the mCpHpH context. Moreover, mCpG and mCpHpG were mainly located in high methylation level (Fig. 2c).

Under continuous cropping stress, when genomic DNA demethylation occurred in both varieties, all the CpG, CpHpG and CpHpH sites in tolerant KX2 were more demethylated than those in sensitive HF55 (Fig. 2a). Specifically, the methylation levels of CpG, CpHpG and CpHpH sites decreased by 6.03, 8.71 and 0.95%, respectively, in tolerant KX2, all of which were higher than those of sensitive HF55 (2.07, 1.90 and 0.77% at CpG, CpHpG and CpHpH sites, respectively). Therefore, the demethylation action of mCpG and mCpHpG contexts may be a more important factor than that of the mCpHpH context in resisting continuous cropping comprehensive stress in soybean.

**Fig. 3:**
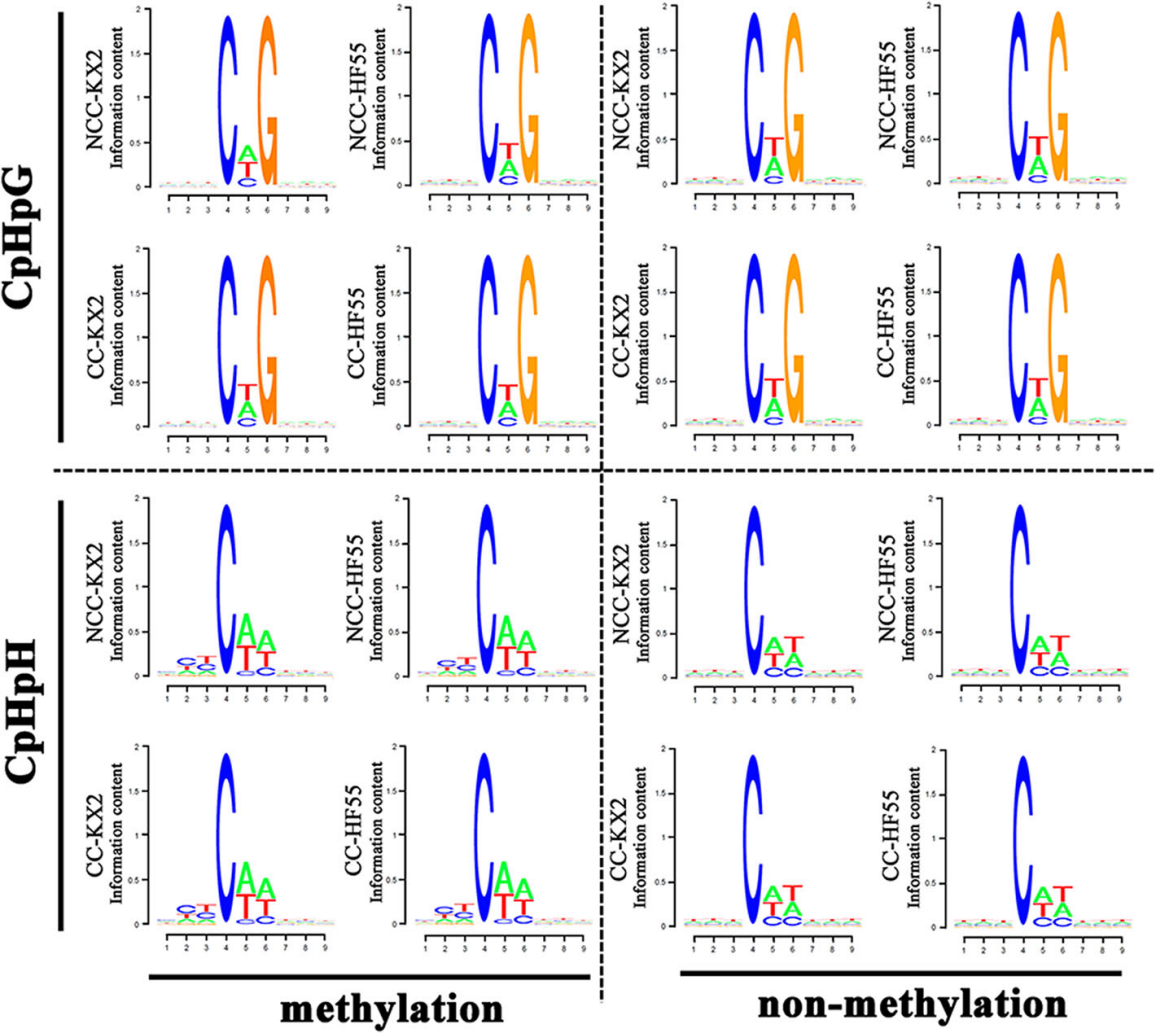
Sequence preferences of methylated and non-methylated CpHpG and CpHpH contexts in two soybean cultivars under different conditions. The methylated cytosine is in the fourth position

Further, we analysed the relationship between CpHpG and CpHpH sequence contexts and methylation preference. We calculated the numbers of 9-mer sequences in which the mC was at the fourth position (Fig. 3). In non-methylation sequence contexts, CpTpG and CpApT were the most frequent, and there was no difference between HF55 and KX2. However, in the methylated CpHpG context, mCpApG was the most frequent in tolerant KX2. Under continuous cropping stress, the mCpApG ratio decreased, while the mCpTpG ratio became dominant, showing that mCpApG demethylation was more important than demethylation of other contexts for the continuous cropping adaptation of tolerant soybean. The methylated CpHpH context was very different from non-methylated sequences. The CpT dinucleotide was the most enriched upstream of mC, followed by ApA, indicating that surrounding sequences may also be important in determining CpHpH methylation.

### Soybean chromosomal DNA demethylation exhibits whole-scale regular characteristics under continuous cropping comprehensive stress

We generated a set of methylation data for each of the twenty chromosomes in the four samples, which revealed the methylation characteristics at the chromosome level. As shown in Table 1, the total methylation levels of C, CpG, CpHpG and CpHpH sites were lowest on chromosome 13 and highest on chromosome 18 in both KX2 and HF55. Moreover, the methylation levels of all chromosomes in tolerant KX2 were lower than those in sensitive HF55. Under continuous cropping stress, cytosine (C, CpG, CpHpG and CpHpH) demethylation occurred on all chromosomes of both tolerant KX2 and sensitive HF55, and tolerant KX2 was more demethylated on all chromosomes than sensitive HF55. Specifically, total C demethylation ranged from 2.68 to 3.56 in KX2 but only from 1.15 to 1.8 in HF55, and chromosome 6 contained the most total C demethylation. In addition, in KX2, the demethylation of CpG and CpHpG contexts (5.22–7.61 and 6.80–10.08, respectively) on all chromosomes was much higher than that of the CpHpH context (0.85–1.02) under continuous cropping stress, and the CpHpG demethylation rate was the highest.

**Table 1:** Methylation characteristics at the chromosome level in sensitive and tolerant soybean varieties under different conditions

Chromosome	NCC-HF55				CC-HF55				NCC-KX2				CC-KX2			
	C	CG	CHG	CHH	C	CG	CHG	CHH	C	CG	CHG	CHH	C	CG	CHG	CHH
Gm01	20.44	69.95	49.21	7.99	18.89	67.44	47.15	7.16	18.71	67.61	46.86	6.60	15.48	61.18	36.83	5.61
Gm02	20.20	68.66	42.62	7.02	18.45	66.33	40.38	6.22	18.28	66.26	39.67	5.69	14.80	60.08	30.14	4.72
Gm03	18.91	64.94	42.91	7.18	17.29	62.48	40.82	6.38	17.40	63.90	41.79	5.92	14.30	57.80	32.71	4.98
Gm04	19.83	67.58	45.87	7.50	18.18	65.04	43.56	6.66	18.03	65.09	43.27	6.15	14.77	58.75	33.47	5.16
Gm05	19.16	64.97	41.15	6.89	17.53	62.61	38.97	6.09	17.37	62.54	38.58	5.61	14.01	55.98	29.27	4.63
Gm06	19.94	67.67	40.73	6.81	18.14	65.16	38.51	6.03	17.49	63.42	36.54	5.45	13.93	56.57	27.56	4.45
Gm07	18.79	64.92	40.52	6.81	17.11	62.29	38.31	6.06	17.06	62.71	38.34	5.60	13.82	56.20	29.48	4.68
Gm08	15.49	57.3	30.66	5.67	14.05	55.07	28.79	4.99	14.08	55.27	29.22	4.65	11.19	48.66	21.82	3.79
Gm09	18.51	63.41	41.90	7.03	16.80	60.48	39.36	6.19	16.48	60.05	38.67	5.64	13.29	53.44	29.36	4.66
Gm10	18.24	63.58	41.28	6.97	16.79	61.89	39.53	6.19	16.51	61.24	38.94	5.72	13.55	55.74	30.19	4.79
Gm11	17.81	59.99	38.69	6.79	16.20	57.57	36.44	5.98	16.01	57.50	36.24	5.50	12.84	51.14	27.13	4.49
Gm12	18.46	62.32	39.60	6.70	16.81	59.46	37.32	5.95	16.96	60.95	37.12	5.44	13.54	53.34	27.98	4.48
Gm13	13.95	50.66	27.08	5.28	12.51	47.87	25.23	4.65	12.34	48.08	24.94	4.24	9.66	41.56	18.14	3.39
Gm14	20.74	70.87	47.16	7.52	19.21	68.86	45.37	6.75	19.28	69.79	46.10	6.29	15.86	63.80	36.02	5.32
Gm15	17.54	62.82	39.20	6.86	15.85	59.60	36.88	6.05	16.10	61.52	37.04	5.59	13.04	54.99	28.09	4.62
Gm16	17.63	63.70	39.28	6.99	16.03	61.26	37.03	6.19	15.94	61.42	37.40	5.68	12.92	55.48	28.30	4.71
Gm17	17.68	58.43	36.47	6.41	16.53	57.88	35.56	5.79	15.51	55.05	32.76	5.23	12.49	49.35	24.67	4.26
Gm18	21.47	73.64	46.09	7.63	19.77	71.30	44.00	6.80	19.43	70.89	43.18	6.25	15.91	64.96	33.36	5.23
Gm19	18.99	66.08	43.02	7.26	17.45	63.90	40.97	6.49	18.05	66.44	42.78	6.11	14.97	61.22	33.45	5.15
Gm20	19.56	67.23	44.25	7.24	17.97	64.98	42.08	6.44	17.78	65.01	41.94	5.96	14.62	58.65	32.71	5.03

**Fig. 4:**
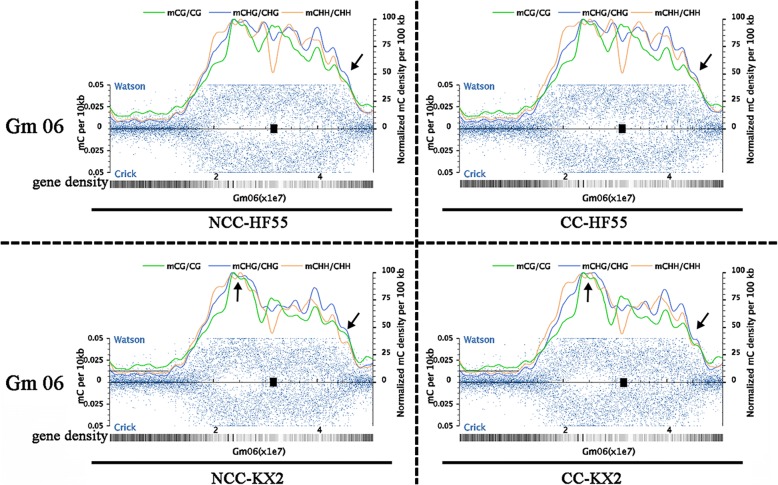
Methylcytosine density throughout chromosome 6 in sensitive HF55 and tolerant KX2 under different conditions. Normalized methylated cytosine over total cytosine positions in 10-kb windows (blue dots, left axis) and normalized methylated CpG, CpHpG, and CpHpH contexts in 100-kb windows (smoothed lines, right axis)

To characterize the distribution of all kinds of C methylation at the chromosome scale in greater detail, we generated dot plots of average C methylation levels in sliding 100-kb windows along each chromosome (Fig. 4, Additional file 1: Figures S2-1–S2-4). Moreover, we acquired the information for gene models and centromere location from the Soybean Genome Browser at SoyBase.org http://soybase.org/gbrowse/cgi-bin/gbrowse/gmax1.01/. [41], and then matched the concrete methylation characteristics in the CpG, CpHpG and CpHpH contexts on all chromosomes. When comparing all kinds of cytosine methylation levels based on the density of genes, we found that DNA methylation was roughly negatively correlated with the density of genes. The density peaks of DNA methylation were most likely to be located in the regions containing few genes, which were mainly located in the pericentromeric region. In contrast, chromosome end regions containing more genes showed the opposite pattern. Further, most chromosomes had a higher methylation level in the telomeric region in the CpG context than in the other two contexts (Additional file 1: Figure S2), suggesting that the methylcytosine distribution at the chromosome level had regional characteristics. However, we did not find obvious, large fluctuations when the methylation distribution was compared between continuous and non-continuous cropping conditions within the same variety, indicating that the demethylation caused by continuous cropping likely occurred at the whole-chromosome scale.

### DNA demethylation induced by continuous cropping comprehensive stress mainly occurs in gene-regulatory regions

**Fig. 5:**
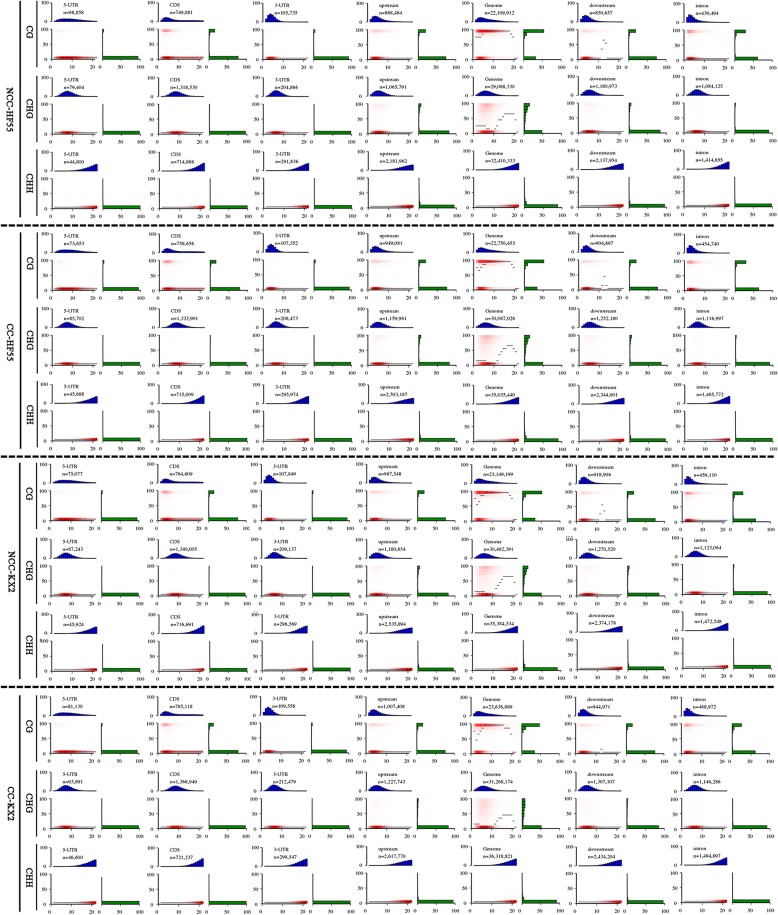
Heat maps of density patterns and methylation distribution of CpG, CpHpG and CpHpH contexts in different genomic regions of two soybean cultivars under different conditions. Each panel represents a separate characteristic, and n represents the number of analysed CpGs, CpHpGs or CpHpHs (per-strand depth ≥ 4) within that feature. X-axis (cytosine density) indicates the number of CpGs, CpHpGs or CpHpHs in 200 bp windows. Y-axis (methylation level) displays the mean methylation level of cytosines in the specific CpGs, CpHpGs or CpHpHs. The black line represents the methylated median value for the specific density of CpGs, CpHpGs or CpHpHs. The red zone reflects the abundance of CpGs, CpHpGs or CpHpHs that fall into bins of given methylation levels and their densities. The top blue bar chart shows the distribution of CpG, CpHpG or CpHpH densities projected onto the horizontal axis of the heat maps. The right green bar chart indicates the distribution of methylation levels, projected onto the vertical axis of the heat maps

To characterize the abundant C methylation in the regions of the soybean genome, we used heat maps to represent the methylation distribution; CpG, CpHpG and CpHpH density distribution; and the relationship between the two in the regions including the 3′-UTR, 5′-UTR, CDS, and upstream, downstream, intron and genome (Fig. 5). We found that in all regions, the densities of CpG, CpHpG and CpHpH contexts increased in that order in the four samples. However, the number of high-methylation-level windows in upstream, downstream, 5′-UTR, 3′-UTR and genome decreased in the order of CpG, CpHpG and CpHpH contexts. Moreover, continuous cropping comprehensive stress also decreased the number of high-methylation-level windows of CpG and CpHpG in these regions in KX2. Therefore, we compared the average methylation density of each context in specific regions. As shown in Table 2, higher mC densities were located upstream (5.75 in KX2 and 6.89 in HF55) and downstream (5.15 in KX2 and 6.09 in HF55) than in other regions, including the genebody, coding region (CDS), 3′-UTR and 5′-UTR. Under continuous cropping stress, total C methylation levels decreased by different degrees in all regions in both varieties, especially in KX2. The levels of upstream, downstream, 5′-UTR and 3′-UTR were reduced more obviously, with reductions of 22.78, 24.08, 23.42, and 21.15%, respectively.

**Table 2:** Relative methylation density (mC/C ratios) in HF55 and KX2 throughout different gene-associated regions under different conditions

Regions	NCC-HF55				CC-HF55				NCC-KX2				CC-KX2			
	C	CG	CHG	CHH	C	CG	CHG	CHH	C	CG	CHG	CHH	C	CG	CHG	CHH
5′-UTR	1.32	3.91	1.37	0.54	1.19	3.75	1.29	0.48	1.11	3.56	1.22	0.40	0.85	3.10	0.82	0.29
CDS	3.33	16.40	2.68	0.79	3.19	16.99	2.57	0.71	3.09	15.94	2.48	0.62	2.60	15.61	1.65	0.44
3′-UTR	1.24	6.38	1.49	0.54	1.12	5.89	1.40	0.49	1.04	5.76	1.34	0.41	0.82	5.02	0.93	0.3
Upstream	6.89	20.87	13.7	3.19	6.11	19.48	12.68	2.81	5.75	19.15	12.2	2.42	4.44	15.73	8.81	1.89
Genebody	4.04	21.12	4.55	1.21	3.74	21.38	4.28	1.07	3.65	20.49	4.24	0.96	2.93	19.37	2.77	0.69
Downstream	6.09	21.63	12.02	2.57	5.42	20.14	11.12	2.28	5.15	19.92	10.78	1.98	3.91	16.34	7.55	1.51

### Some vital metabolism processes and related DMGs in resisting continuous cropping comprehensive stress are revealed by DMG analysis

**Fig. 6:**
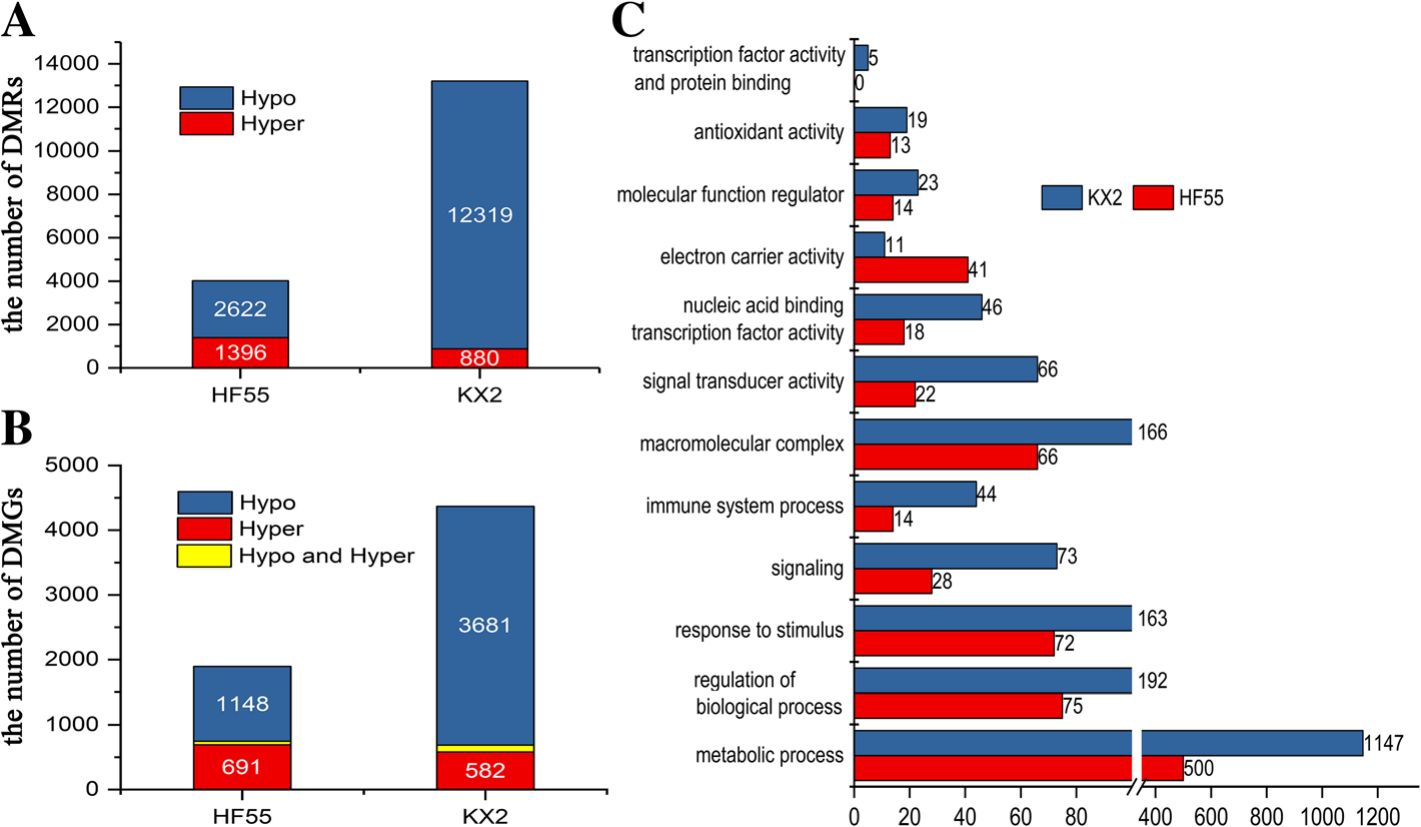
Statistical analysis of differential methylation in sensitive HF55 and tolerant KX2 under different conditions. **a** Number of differentially (hypo- and hyper-) methylated regions (DMRs); **b** Number of differentially (hypo-, hyper-, hypo- and hyper-) methylated genes (DMGs); **c** Gene Ontology (GO) categories significantly enriched in the DMRs related to stress

To investigate differential methylation under continuous cropping stress, we identified differentially methylated regions (DMRs), which denote genomic regions of adjacent CpG, CpHpG or CpHpH sites that are differentially methylated. DMRs were identified in windows containing at least five CpG (or CpHpG or CpHpH) sites at the same position in two sample genomes. A total of 13,199 DMRs in tolerant KX2 and 4018 DMRs in sensitive HF55 were identified, and the hypomethylated proportion in KX2 (95.33%) was much higher than that in HF55 (65.26%) (Fig. 6a). A number of DMGs were identified in KX2 (4475) and HF55 (1951), which consisted of hypomethylated genes and hypermethylated genes. In KX2, the number of hypomethylated genes was significantly higher (5.67 times) than that of hypermethylated genes under continuous cropping stress, whereas the numbers of hypo- and hypermethylated genes were not very different in HF55 (only 1.61 times) (Fig. 6b). GO annotation revealed that these DMGs are involved in diverse biological processes, such as metabolic processes, response to stimulus, signal, transcription, macromolecular complex and biological regulation (Fig. 6c).

**Fig. 7:**
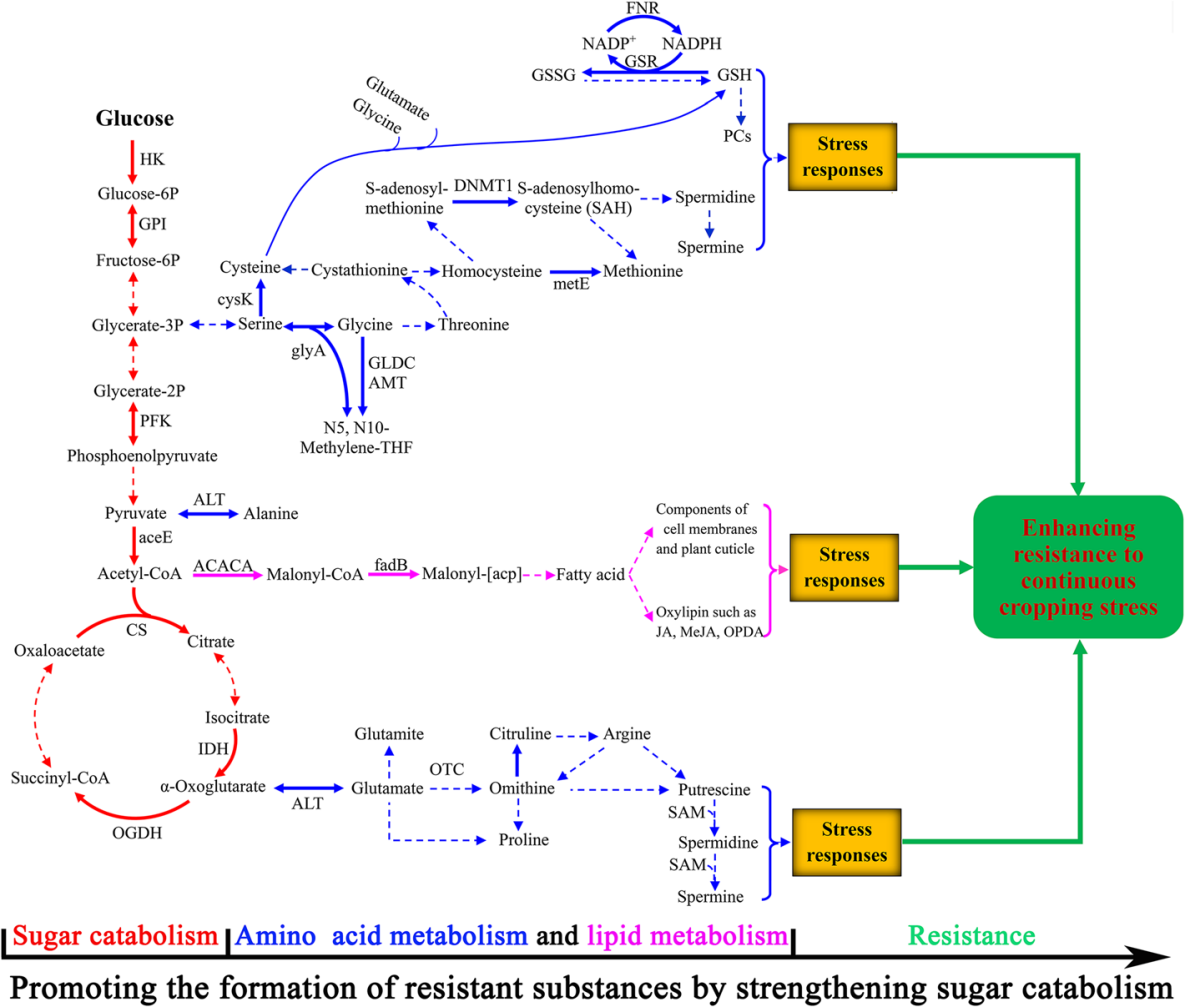
Primary metabolic pathways that respond to continuous cropping comprehensive stress. Solid lines represent one-step reactions and corresponding differentially demethylated enzyme genes. Dotted lines indicate that enzyme genes are not differentially demethylated and/or multi-step reactions. HK: hexokinase; GPI: glucose-6-phosphate isomerase; PFK: 6-phosphofructokinase 1; aceE: pyruvate dehydrogenase E1 component; CS: citrate synthase; IDH: isocitrate dehydrogenase; OGDH: 2-oxoglutarate dehydrogenase E1 component; ALT: alanine transaminase; cysK: cysteine synthase A; glyA: glycine hydroxymethyltransferase; GLDC: glycine dehydrogenase; AMT: aminomethyltransferase; metE: 5-methyltetrahydropteroyltriglutamate-homocysteine methyltransferase; DNMT1: DNA (cytosine-5)-methyltransferase 1; OTC: ornithine carbamoyltransferase; ACACA: acetyl-CoA carboxylase; fabD: [acyl-carrier-protein] S-malonyltransferase; GSR: glutathione reductase (NADPH); FNR: ferredoxin-NADP(+) reductase; PCs: Phytochelatins; JA: jasmonic acid; MeJA: methyl JA; OPDA: 12-oxo-10,15(Z) phytodienoic acid

Detailed pathway-based analyses showed that DMGs induced by continuous cropping comprehensive stress in tolerant KX2 are mainly involved in the processes of glucose, amino acid and fatty acid metabolism. As shown in Fig. 7 and Table 3, the *HK*, *aceE*, *CS*, *IDH*, and *OGDH* genes encode hexokinase, pyruvate dehydrogenase E1 component, citrate synthase, isocitrate dehydrogenase and 2-oxoglutarate dehydrogenase E1 component, respectively, which are the key enzymes for glucose catabolism. Glucose catabolism can supply energy, reductants and some materials for amino acid and fatty acid synthesis. The amino acid metabolism-related genes *ALT*, *glyA*, *cysK*, *metE*, *DNMT1*, and *OTC* encode alanine transaminase, glycine hydroxymethyltransferase, cysteine synthase A, 5-methyltetrahydropteroyltriglutamate-homocysteine methyltransferase, DNA (cytosine-5)-methyltransferase 1, and ornithine carbamoyltransferase, respectively. These enzymes may catalyse glutamate, glycine, cysteine, methionine, S-adenosylhomo-cysteine (SAH) and citrulline synthesis, which will contribute to the synthesis of polyamines (putrescine, spermidine, and spermine), GSH and phytochelatins (PCs). The *GSR* and *FNR* genes encode glutathione reductase (NADPH) and ferredoxin-NADP(+) reductase, which can enhance GSH regeneration. GSH is actively resistant to stress because it is an important antioxidant, which can protect tissues from peroxide damage. Further, the genes *glyA*, *GLDC*, and *AMT* can catalyse one-carbon-unit N5,N10-methylene-THF synthesis from Ser and Gly, which are involved in nucleotide synthesis. The *ACACA* and *fadB* genes encode acetyl-CoA carboxylase and [acyl-carrier-protein] S-malonyltransferase, which will enforce the synthesis of fatty acids and their derivatives, such as jasmonic acid (JA), methyl JA (MeJA) and 12-oxo-10,15(Z) phytodienoic acid (OPDA). These compounds are all associated with plant resistance to biotic and abiotic stresses. All of these genes were differentially demethylated in tolerant KX2 but not in sensitive HF55 under continuous cropping stress.

**Table 3:** Stress-induced DMGs involved in metabolism processes and their demethylated types in soybean

Abbreviation	Full name of enzyme	Gene name in Soybean	DMG Type
HK	hexokinase [EC:2.7.1.1]	Glyma07g12190	CG
GPI	glucose-6-phosphate isomerase [EC:5.3.1.9]	Glyma19g02031	CHG
PFK	6-phosphofructokinase 1 [EC:2.7.1.11]	Glyma18g22780	CHG
aceE	pyruvate dehydrogenase E1 component [EC:1.2.4.1]	Glyma07g03930; Glyma17g03560	CHG; CHG
CS	citrate synthase [EC:2.3.3.1]	Glyma18g12393	CHG
IDH	isocitrate dehydrogenase [EC:1.1.1.42]	Glyma08g17080	CHG
OGDH	2-oxoglutarate dehydrogenase E1 component [EC:1.2.4.2]	Glyma17g03560	CG/CHG; CHG
ACACA	acetyl-CoA carboxylase [EC:6.4.1.2 6.3.4.14 2.1.3.15]	Glyma04g11550	CHG
fabD	[acyl-carrier-protein] S-malonyltransferase [EC:2.3.1.39]	Glyma18g06500	CG
ALT	alanine transaminase [EC:2.6.1.2]	Glyma02g04320	CG
glyA	glycine hydroxymethyltransferase [EC:2.1.2.1]	Glyma18g27710	CHG
GLDC	glycine dehydrogenase [EC:1.4.4.2]	Glyma17g34690	CG
AMT	aminomethyltransferase [EC:2.1.2.10]	Glyma15g11590	CG
cysK	cysteine synthase A [EC:2.5.1.47]	Glyma02g15640; Glyma15g41600	CHG; CHG
metE	5-methyltetrahydropteroyltriglutamate-homocysteine methyltransferase [EC:2.1.1.14]	Glyma17g23730	CHG
DNMT1	DNA (cytosine-5)-methyltransferase 1 [EC:2.1.1.37]	Glyma11g08861; Glyma01g01120; Glyma16g17720	CHG; CG; CG/CHG
OTC	ornithine carbamoyltransferase [EC:2.1.3.3]	Glyma06g03361	CG
GSR	glutathione reductase (NADPH) [EC:1.8.1.7]	Glyma02g08180	CHG
FNR	ferredoxin-NADP(+) reductase [EC 1.18.1.2]	Glyma08g17080	CHG

**Fig. 8:**
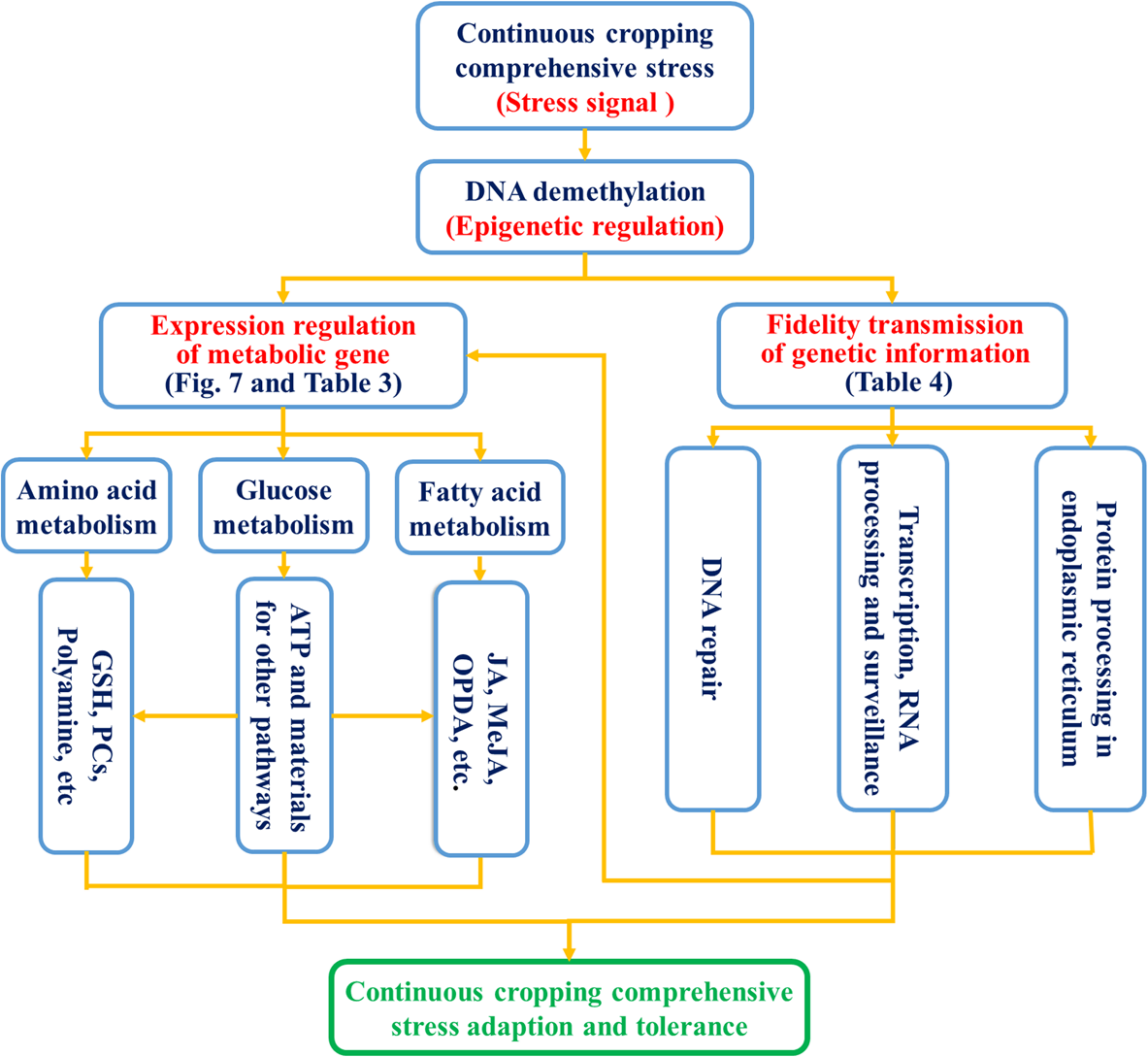
Schematic showing the points at which soybean copes with continuous cropping comprehensive stress by metabolic and genetic regulation resulting from DNA demethylation. The continuous cropping stress signal resulted in DNA demethylation. Some metabolic and genetic processes were modified, causing the plants to adapt to the stress in the continuous cropping environment

In addition, based on GO annotation, some stress-response genes are involved in the processes of DNA repair (MutLα, XPD, and TFIIH4), RNA surveillance (PP2A and RNGTT), spliceosome formation (U5 snRNA) and protein processing in the endoplasmic reticulum (P97) (Fig. 8 and Table 4). These processes are closely related to plant adaptability to stress. Moreover, these DMGs involved in metabolism processes and fidelity transmission of genetic information were characterized with demethylation of CpG and CpHpG contexts (Table 3 and Table 4).

**Table 4:** DMGs involved in fidelity transmission of genetic information within GO annotation and their demethylated types in soybean

Process involved	Pathway involved	Transcript name	Protein name	DMG Type
DNA repair	Nucleotide excision repair	Glyma16g28290	XPD	CG
		Glyma07g31520	TFIIH4	CG
	Mismatched repair	Glyma10g02390	MutLα	CG
RNA biosynthesis	Basal transcription factors	Glyma07g31520	TFIIH4	CG
		Glyma16g28290	XPD	CG
	Spliceosome	Glyma17g11410	Snu114	CHG
	RNA surveillance	Glyma03g34240	PP2A	CHG
		Glyma03g14700; Glyma01g27240	RNGTT	CG; CHG
Protein biosynthesis	Protein processing in endoplasmic reticulum	Glyma06g19000; Glyma03g33990; Glyma12g30060	P97	CG; CG; CHG

## Discussion

DNA methylation changes in plant genomes can generate novel and heritable phenotypic variation, which will improve tolerance, resistance and adaptation to poor environments by influencing gene expression [42, 43]. The striking phenotypic difference between tolerant KX2 and sensitive HF55 prompted us to profile their methylomes in response to continuous cropping stress. The high-resolution analysis provided unique insight into the plasticity of DNA methylation in response to the comprehensive stress of continuous cropping. We observed that the total methylation ratios of tolerant KX2 and sensitive HF55 were 16.78 and 18.57%, respectively, which are lower than those of both maize (20%) [35] and rice (24.3%) [44] but more than three times that of Arabidopsis (5.26%) [45], indicating specific methylation levels among diverse plant species [29]. Lukens and Zhan reported that a moderate cytosine methylation level played an important role in maintaining genome stability, contributing to the silencing of transposable elements and controlling the transcription of some genes in plants [42]. In this study, we found that continuous cropping comprehensive stress induced genomic DNA demethylation in soybean. The tolerant variety KX2 was able to rapidly reduce its DNA methylation level (by 18.77%), while the sensitive variety HF55 showed a low ability to adjust its DNA methylation level (decrease of 8.35%) upon exposure to continuous cropping stress, suggesting a link between the plasticity of DNA methylation and plant tolerance performance. A decrease in DNA methylation level will cause the activation of some genes, which will enhance the expression of stress-resistant proteins [32, 46–48]. Therefore, we infer that the tolerant variety KX2 can better adapt to the comprehensive stress of continuous cropping than the sensitive variety HF55 through further demethylation. This finding is in agreement with earlier results on DNA demethylation in response to adverse conditions, such as biotic stress [32, 49, 50], salt stress [51] and Fe deficiency [52]. The reason is that chromatin demethylation and the relaxation of its structure could serve as a transcriptional switch for many stress-regulated genes [15]. However, hypermethylation of cytosine residues has also been uncovered in pea root tips in response to water deficit stress [53] and in tobacco [54] and potato [55] cell cultures in response to osmotic stress. Therefore, epigenetic change at the DNA methylation level, whether hypomethylation or hypermethylation, plays an important role in plant adaptation to environmental stress and growth and development. Importantly, though, distinct epigenetic responses occur between different plants and in response to different stress stimuli.

DNA methylation in plants occurs in three cytosine contexts including CpG, CpHpG and CpHpH (H replacing A, C or T) [56, 57], and it appears to have various functions, including regulating the expression of some genes, reprogramming and imprinting [58]. The cytosine methylation patterns result not only from the establishment and maintenance of mCs but also from demethylation [59]. The removal of methylcytosine can be accomplished via passive or active processes. In passive demethylation, the mCs are replaced with unmethylated cytosines during DNA replication, while in active demethylation, the methyl mark is removed by 5mC glycosylases such as DME, DML and ROS1. These enzymes, possessing both glycosylase (base excision) and AP lyase activity that are directed towards mCs, are involved in base excision repair [60]. In our work, the relative expression levels of DML and ROS1 in KX2 were significantly higher than those in HF55 under continuous cropping stress. The high expression of these enzymes should be one of the factors causing extensive demethylation in genomic DNA [25]. Active DNA demethylation is important in maintaining epigenomic plasticity to enable efficient response to environmental stresses in a timely manner [17, 59]. These results further confirm that the DNA demethylation is related to the resistance of soybean to the continuous cropping comprehensive stress. Therefore, how to apply demethylase in plant resistance to this kind of stress should be researched in the future.

Cytosine bases of the nuclear genome in higher plants are often extensively methylated [40, 56]. In this work, we described the profile of DNA methylation density for each of the 20 chromosomes in 100-kb windows. Interestingly, chromosome end regions showed lower DNA methylation density, where gene density was high, suggesting that cytosine methylation occurred in the intergenic regions. Figure 4 and Additional file 1: Figure S2(-1,-2,-3,-4) also show low methylation density in the telomeric regions. These results are in agreement with previous reports on Arabidopsis telomere methylation. Vaquero-Sedas et al. found that Arabidopsis telomeres have lower levels of DNA methylation than internal transcribed spacers or subtelomeres [61]. Later, Vega-Vaquero et al. confirmed that DNA methylation is indeed absent in Arabidopsis telomeres based on experiments on high telomeric C-rich strand production efficiencies and methylation-dependent restriction enzyme analyses [62]. In addition, they pointed out that the degree of telomeric DNA denaturation during the process of sequencing or the formation of telomeric C-rich strand secondary structures such as the i-motif might cause the overestimation of telomeric methylation [63, 64]. Schoeftner and Blasco also considered that conserved telomeric repeats remained unmethylated because the asymmetric target units (CCCTAAA) n in plants lack methylable cytosines [65].

In mammals, DNA methylation predominantly occurs at cytosines in CpG sequences, while in plants, methylation of CpH sequences (CpHpG and CpHpH, where H can be A, C or T) is also present and involved in epigenetic regulation and gene expression [66, 67]. Therefore, detecting the context proximal to sites of CpH methylation is essential for determining whether stresses caused some enrichments of particular local sequences and basal changes, as previously reported in Arabidopsis DNA methylomes [45, 68]. No local sequence enrichment was observed upstream of mCpHpG sites, while the base following the methylcytosine tended to be adenine or thymine (mCpA/TpG) (Fig. 3). This trend is consistent with a nearest-neighbour analysis of wheat germ DNA that found a higher level in mCpApG and mCpTpG sites than in mCpCpG sites [69]. Further, we found that the base preference following methylcytosine in mCpHpG changed under continuous cropping comprehensive stress in tolerant KX2, and mCpTpG became more common than mCpApG. Therefore, we infer that mCpApG sequences are more easily demethylated than other sequences under continuous cropping comprehensive stress in tolerant KX2, which resulted in the decrease in the percentage of mCpApG and thus the dominance of mCpTpG. This process may regulate gene expression, but further research is necessary to clarify the role of CHG demethylation, as reviewed by Pelizzola and Ecker [70]. In addition, similar to the mCpHpH context, mC also tended to be followed by an A or a T. This result is consistent with data from mammalian and Arabidopsis genomes indicating that mCpT and mCpA sequences are more frequent than mCpC sequences [45, 68, 71].

DMRs, GO annotation and KEGG pathway analysis indicated that some DMGs in response to continuous cropping comprehensive stress are involved in some vital metabolic processes (Fig. 6). *HK*, *aceE*, *CS*, *IDH*, and *OGDH* are key genes for glucose catabolism, which is the central metabolic pathway in all organisms. This pathway produces ATP, reductants and some materials for amino acid and fatty acid synthesis. Additionally, glucose catabolism is directly involved in adaptation of plants to environmental stresses, such as nutrient limitation, drought, low-temperature and osmotic stress [72]. The *ALT*, *glyA*, *cysK*, *metE*, *DNMT1*, and *OTC* gene products catalyse the synthesis of some important amino acids such as SAH, methionine, cysteine, glycine, glutamate and citrulline. These amino acids are necessary for the biosynthesis of some defence compounds, such as GSH, polyamines and PCs. The *GSR* and *FNR* gene products also enhance GSH regeneration. GSH has many distinct functions in plant stress defence, including removing harmful H_2_O_2_ to protect tissues from peroxide damage, controlling gene expression linked to the redox state of cells and being an important reducing cofactor of many enzymes related to reactive oxygen species (ROS) detoxification [73–75]. Some researchers reported that plants exposed to salicylic acid (SA) (which plays a key role in plant stress tolerance) and abscisic acid (ABA) (which is related to environmental stress adaptation) exhibited higher GSH concentrations and glutathione reductase (GR) activity than those without such exposure, which further certifies the relationship between GSH content and stress defence [76–78]. Moreover, GSH is also related to the synthesis of PCs, which is involved in stress response [78]. In addition, SAH is the product catalysed by DNMT1 in the S-adenosylmethionine/homocysteine cycle. Fuso and Lu et al. reported that SAH is a strong DNA methyltransferase inhibitor that will reinforce genomic DNA hypomethylation [79, 80]. Polyamine synthesis is an important nitrogen-metabolizing pathway regulating ammonia metabolism and organic nitrogen balance in plant cells [81–85]. During the process of polyamine generation, some intermediates, such as nitric oxide (NO) and γ-aminobutyric acid (GABA), can also be produced [86, 87]. Polyamines, NO and GABA all play important roles in the regulation of plant development and as signal molecules mediating some responses to biotic and abiotic stressors, including pathogens, heavy metal, drought and salt [85, 88–91]. The *ACACA* and *fadB* genes contribute to fatty acid synthesis. Fatty acids not only are crucial components of cellular membranes, suberin, and cutin waxes, but also are important for the remodelling of membrane fluidity, which will increase plant resistance to drought, physical injury and infection by pathogenic microorganisms [92, 93]. Moreover, fatty acids are the materials for the synthesis of substances related to environmental stress adaptation, including JA, MeJA and OPDA, which are also associated with plant basal immunity and responses to pathogens [94, 95]. Therefore, based on the analysis above and our results, we think these substances are involved in the response to continuous cropping comprehensive stress, including biological barriers, allelopathic autotoxicity, the deterioration of soil physicochemical properties and soil fertility imbalance, and DNA demethylation may be the source inducing these complex resistance mechanisms.

The stable inheritance of DNA genetic information, RNA transcription and correct protein synthesis are vital molecular processes for ensuring cell-cycle progression and various biofunctions, all of which are involved in plant growth and stress response [96–99]. In this work, we observed that some DMGs in tolerant KX2 were involved in continuous cropping comprehensive stress responses (GO annotation). These genes took part in the processes of DNA repair, transcription, RNA splicing, RNA surveillance, and protein processing in the endoplasmic reticulum (Fig. 8, Table 4). These molecular processes are capable of adjusting the levels of mRNA and functional proteins that will alter some metabolic processes and thus participate in the stress response [99–101]. Consequently, resistant plants can tolerate complicated environmental stresses more efficiently and effectively through metabolic networks and fidelity transmission of genetic information caused by DNA methylation changes, enhancing their adaptability under continuous cropping comprehensive stress (both biotic and abiotic stresses).

## Conclusion

Genomic DNA demethylation was closely related to soybean adaptability to the continuous cropping comprehensive stress, which was further verified by the increased expression of DNA demethylases ROS1 and DML. The demethylation of mCpG and mCpHpG (mCpApG preferred) contexts was more important, which mainly occurred in gene-regulatory regions at whole-chromosome scale. Among the DMGs, GO annotation and KEGG pathway analysis further demonstrated that various stress responders generated through strengthened glucose catabolism, amino acid and fatty acid anabolism, as well as fidelity transmission of genetic information, played important roles in soybean adaptability to this kind of adversity.

## Methods

### Plant materials and growing conditions

The soybean cultivars used in this study included the continuous cropping-tolerant variety KX2 (breeding and provision by Daqing Branch of Heilongjiang Academy of Agriculture Science) and continuous cropping-sensitive variety HF55 (provision by Genetic Breeding Laboratory of Agricultural College of Heilongjiang Bayi Agricultural University; breeding by Hejiang Institute of Agricultural Sciences, Heilongjiang Academy of Agricultural Sciences). Continuous cropping soil was collected from Lindian County, Heilongjiang Province, China, where soybean has been cultivated continuously for 6 years. The control included neighbouring non-continuous cropping soil with physicochemical properties and fertility similar to those of the continuous cropping soil. Soybean seeds were planted in pots (diameter 25 cm, depth 20 cm) filled with continuous cropping or control soil and exposed to 25/18 °C (day/night) and 70% relative humidity for 60 days in a greenhouse. Light, temperature and humidity conditions remained constant throughout the experimental periods. Soybean plants were collected carefully after 60 days of sowing. Four plants per replicate were used for measuring plant height, total leaf area and nodule number. The roots, stems, leaves and nodules of those four soybean plants were packaged in draft paper, oven-dried at 105 °C for 30 min, and then dried at 80 °C to a constant weight. The total leaf area was determined through the dry weight ratio method. Chlorophyll content from leaf tissues was measured from different positions on two of the uppermost, youngest, fully expanded leaves using a portable chlorophyll content meter, CCM-200 PLUS (Opti-sciences, USA). Three repetitions per treatment were used.

### Whole-genome bisulfite sequencing library construction and sequencing

Leaf material was excised from the uppermost third of the functional leaves of 60-d-old plants, frozen in liquid nitrogen and stored at − 80 °C until DNA isolation. There were three repetitions per treatment. Genomic DNA was extracted using a QIAamp DNA Micro Kit (Qiagen, USA) according to the manufacturer’s instructions. DNA concentration was quantified using a Qubit Fluorometer (Invitrogen, USA), and DNA integrity was detected by 1% agarose gel electrophoresis. After that, DNA of three repetitions was mixed in equal amounts for library construction.

For normal WGBS library construction, the DNA was fragmented to 100–300 bp by sonication using a Bioruptor (Diagenode, Belgium), following blunt-ending, addition of dA to the 3′-end, and ligation of adaptors to protect against bisulfite conversion. DNA fragments were treated with sodium bisulfite using a Zymo EZ DNA Methylation-Gold kit (Zymo Research, USA). After sodium bisulfite conversion, unmethylated cytosine residues are converted to uracil, whereas 5-methylcytosine (5mC) remains unchanged. Fragments with different insert sizes were excised from the same lane of a 2% TAE agarose gel. Products were purified by using a QIAquick Gel Extraction kit (Qiagen, USA) and amplified by PCR. After PCR amplification, uracil residues were converted to thymine. Finally, the qualified library was sequenced using a HiSeq 2000 platform.

### Data analysis

After sequencing, the raw reads were filtered by removing adaptor sequences and low-quality reads using the Illumina analysis pipeline. During this process, due to bisulfite conversion of cytosine to uracil, cytosines on the coding strand were changed to thymidines, and guanines on the template strand were changed to adenosines. Then, the clean and high-quality reads were aligned to the reference genome Phytozome v9.0 (https://genome.jgi.doe.gov/portal/pages/dynamicOrganismDownload.jsf?organism=Phytozome) using SOAP 2.20 software. Only perfectly matched reads were used for methylation analysis. Methylation level was determined by dividing the number of reads covering methylated cytosine (mC) by the total reads covering cytosine (mC/C) [102, 103]. The ratio of mCpG, mCpHpG, or mCpHpH to total mC was used to calculate the proportion of mCpG, mCpHpG, or mCpHpH at all mC sites, respectively. DMRs were identified by comparing the methylation level difference in the same sliding window of genomes between continuous cropping samples and noncontinuous cropping samples. Only windows that contained at least five CpG (or CpHpG or CpHpH) sequences were used, and changes in methylation level after continuous cropping stress had to be at least 2-fold. *P*-values associated with DMRs were calculated by Fisher’s exact test, and *P* values < 0.05 were considered significant. All adjacent differentially methylated windows were collapsed into a single DMR. Genes containing DMRs in the genebody and/or 2-kb flanking sequences were considered DMGs. GO enrichment analysis was performed using the BiNGO tool to analyse the molecular functions of DMGs. P-values ≤0.05 after family-wise error rate correction were considered significantly enriched. KEGG pathways were used to analyse the biochemical metabolic processes that DMGs were involved in.

### Expression analysis of demethylase genes

Total RNA was extracted from leaves of tolerant KX2 and sensitive HF55 soybean using an RNA extraction kit (UNIQ-10, SK1321, Sangon Biotech, China). The RNA concentration was calculated by measuring the absorbance at 260 nm, and the purity was evaluated by the ratios of 260/280 nm and 260/230 nm using a NanoDrop spectrophotometer (NanoDrop Technologies, USA). cDNA was synthesized using RevertAid Premium Reverse Transcriptase (Thermo Scientific™ EP0733) with 6-mer random primers as recommended. The synthesized cDNA was subjected to qPCR. Specific primers were designed using Primer Premier 5.0 software (Premier Biosoft International, Palo Alto, USA) (Additional file 1: Table S4). The *ACTIN* gene was used as a reference gene to normalize the amount of RNA in each sample. The qPCR was performed in an ABI StepOne Plus instrument in a 20 μL reaction mixture containing 2 μL cDNA, 4 μmol forward and reverse primers, and 10 μL SybrGreen qPCR Master Mix (Takara Biotech, China). Thermal cycling conditions were as follows: pre-incubation at 95 °C for 3 min, followed by 45 cycles of denaturation at 95 °C for 7 s, annealing at 57 °C for 10 s and elongation at 72 °C for 15 s. A melting curve was analysed at the end of the qPCR to verify specific amplification. The relative expression quantity was determined by the 2^–ΔΔCt^ method, where ΔCt = (Ct _target gene_ − Ct _actin gene_) and ΔΔCt = (ΔCt _treatment_–ΔCt _control_). The experimental data were statistically analysed with the t test using SPSS software (version 25.0, SPSS Inc., USA). *P* values lower than 0.05 were considered statistically significant.

## Additional file

Additional file 1: **Table S1.** Morphological indexes of soybean plants under different conditions. **Table S2.** Effective coverage of each chromosome in each sample. **Table S3.** Effective coverage of various regions in each sample. **Table S4.** The primer sets used in qRT-PCR. **Figure S1.** Cumulative distribution of effective sequencing depth in total cytosine and three sequence contexts. **Figure S2-1.** Methylcytosine density throughout chromosome one to five in sensitive HF55 and tolerant KX2 under different conditions. **Figure S2-2.** Methylcytosine density throughout chromosome six to ten in sensitive HF55 and tolerant KX2 under different conditions. **Figure S2-3.** Methylcytosine density throughout chromosome eleven to fifteen in sensitive HF55 and tolerant KX2 under different conditions. **Figure S2-4.** Methylcytosine density throughout chromosome sixteen to twenty in sensitive HF55 and tolerant KX2 under different conditions. (DOCX 10182 kb)

## Abbreviations

5mC: Cytosine methylation
ABA: Abscisic acid
CMT: Chromomethylase 3
DME: Demeter
DMGs: Differentially methylated genes
DML: Demeter-like
DMRs: Differentially methylated regions
DRM2: Domains Rearranged Methyltransferase 2
GABA: γ-aminobutyric acid
GO: Gene Ontology
GR: Glutathione reductase
HF55: He Feng 55
JA: Jasmonic acid
KEGG: Kyoto Encyclopedia of Genes and Genomes
KX2: Kang Xian 2
MeJA: Methyl JA
MET1: DNA methyltransferase 1
NO: Nitric oxide
OPDA: 12-oxo-10,15(Z) phytodienoic acid
PCs: Phytochelatins
qRT-PCR: Real-time quantitative PCR
RdDM: RNA-directed DNA methylation
ROS: Reactive oxygen species
ROS1: Repressor of Silencing 1
SA: Salicylic acid
SAH: S-adenosylhomo-cysteine
siRNAs: Small interfering RNAs
WGBS: Whole-genome bisulfite sequencing
