# Supplementary material for: High-resolution DNA methylome reveals that demethylation enhances adaptability to continuous cropping comprehensive stress in soybean
Source: BMC Plant Biol. 2019 Feb 18;19:79. doi: 10.1186/s12870-019-1670-9 (PMC6380062; doi:10.1186/s12870-019-1670-9)
Supplement: Supplementary file 1 — Table S1. Morphological indexes of soybean plants under different conditions. Table S2. Effective coverage of each chromosome in each sample. Table S3. Effective coverage of various regions in each sample. Table S4. The primer sets used in qRT-PCR. Figure S1. Cumulative distribution of effective sequencing depth in total cytosine and three sequence contexts. Figure S2-1. Methylcytosine density throughout chromosome one to five in sensitive HF55 and tolerant KX2 under different conditions. Figure S2-2. Methylcytosine density throughout chromosome six to ten in sensitive HF55 and tolerant KX2 under different conditions. Figure S2-3. Methylcytosine density throughout chromosome eleven to fifteen in sensitive HF55 and tolerant KX2 under different conditions. Figure S2-4. Methylcytosine density throughout chromosome sixteen to twenty in sensitive HF55 and tolerant KX2 under different conditions. (DOCX 10182 kb) [file 12870_2019_1670_MOESM1_ESM.docx]

**Supplementary Figure and Table**

**Table S1 Morphological indexes of soybean plants under different conditions**

| **Sample**  **name** | **Height (cm)**  **/plant** | **Total leaf area**  **(cm^2^)/4plants** | **Stem and leaf dry weight (g)/4plants** | **Root dry weight (g)/4plants** | **Nodule Number**  **/4plants** | **Nodule dry weight (g)/4plants** | **Total chlorophyll content（CCI）** |
| --- | --- | --- | --- | --- | --- | --- | --- |
| NCC-HF55 | 39.05±2.25 | 1529.60±362.07 | 7.90±0.92 | 3.18±0.34 | 220.33±7.32 | 0.58±0.08 | 21.22±0.54 |
| CC-HF55 | 32.86±2.31***** | 483.40±55.45***** | 4.52±0.51***** | 2.98±0.53 | 14.67±7.32***** | 0.02±0.01***** | 11.22±2.41***** |
| NCC-KX2 | 41.23±3.39 | 1969.32±243.00 | 9.05±1.26 | 2.03±0.44 | 137.00±51.59 | 0.66±0.17 | 18.25±2.10 |
| CC-KX2 | 40.64±1.74 | 1771.79±487.90 | 8.82±1.07 | 2.41±0.35 | 210.67±101.46 | 0.96±0.35 | 16.74±1.47 |

**The data are mean ± SD (n = 3). The analysis was performed using an independent samples t-test. Symbol (*) indicates a significant difference at P＜0.05 between NCC and CC of the same species. NCC: non-continuous cropping; CC: continuous cropping.**

**Table S2 Effective coverage of each chromosome in each sample**

| **Chromosomes** | **NCC-HF55** | | | | **CC-HF55** | | | | **NCC-KX2** | | | | **CC-KX2** | | | |
| --- | --- | --- | --- | --- | --- | --- | --- | --- | --- | --- | --- | --- | --- | --- | --- | --- |
|  | C | CG | CHG | CHH | C | CG | CHG | CHH | C | CG | CHG | CHH | C | CG | CHG | CHH |
| **Gm01** | 89.56 | 89.48 | 90.18 | 89.48 | 89.66 | 88.95 | 89.78 | 89.72 | 89.88 | 89.53 | 90.30 | 89.86 | 91.24 | 90.63 | 91.39 | 91.29 |
| **Gm02** | 90.17 | 90.07 | 91.19 | 90.03 | 90.32 | 89.29 | 90.86 | 90.37 | 89.98 | 89.38 | 90.57 | 89.97 | 91.29 | 90.42 | 91.65 | 91.34 |
| **Gm03** | 83.36 | 82.75 | 83.93 | 83.34 | 83.07 | 81.71 | 83.04 | 83.23 | 86.76 | 86.36 | 87.07 | 86.76 | 88.40 | 87.73 | 88.49 | 88.47 |
| **Gm04** | 89.47 | 89.77 | 90.37 | 89.3 | 89.76 | 89.50 | 90.25 | 89.71 | 89.99 | 90.07 | 90.64 | 89.88 | 91.58 | 91.41 | 92.00 | 91.54 |
| **Gm05** | 89.18 | 89.23 | 89.81 | 89.07 | 89.44 | 88.86 | 89.58 | 89.49 | 89.59 | 89.31 | 89.90 | 89.58 | 91.02 | 90.50 | 91.10 | 91.07 |
| **Gm06** | 89.01 | 88.98 | 89.74 | 88.91 | 89.46 | 88.89 | 89.70 | 89.49 | 86.45 | 85.45 | 86.71 | 86.53 | 87.73 | 86.46 | 87.72 | 87.88 |
| **Gm07** | 87.79 | 86.67 | 88.48 | 87.82 | 88.02 | 86.25 | 88.21 | 88.21 | 89.21 | 88.28 | 89.62 | 89.27 | 90.83 | 89.72 | 91.04 | 90.93 |
| **Gm08** | 90.35 | 89.97 | 91.24 | 90.26 | 90.87 | 89.84 | 91.25 | 90.93 | 91.15 | 90.40 | 91.64 | 91.17 | 92.49 | 91.57 | 92.77 | 92.56 |
| **Gm09** | 89.17 | 89.26 | 89.95 | 89.05 | 89.07 | 88.4 | 89.22 | 89.12 | 88.80 | 88.44 | 89.04 | 88.81 | 90.31 | 89.66 | 90.33 | 90.38 |
| **Gm10** | 87.55 | 87.45 | 88.50 | 87.42 | 87.99 | 87.36 | 88.47 | 88.00 | 87.38 | 87.03 | 87.95 | 87.34 | 88.99 | 88.29 | 89.26 | 89.04 |
| **Gm11** | 90.32 | 90.57 | 91.20 | 90.15 | 90.74 | 90.42 | 91.19 | 90.71 | 90.77 | 90.66 | 91.32 | 90.70 | 92.15 | 91.84 | 92.50 | 92.14 |
| **Gm12** | 88.31 | 87.65 | 88.92 | 88.3 | 88.65 | 87.36 | 88.78 | 88.78 | 88.82 | 87.92 | 89.13 | 88.88 | 90.23 | 89.03 | 90.30 | 90.37 |
| **Gm13** | 88.43 | 84.44 | 88.53 | 88.88 | 89.17 | 84.64 | 88.78 | 89.77 | 90.41 | 86.05 | 89.97 | 90.99 | 91.47 | 86.82 | 90.86 | 92.12 |
| **Gm14** | 85.78 | 84.77 | 86.23 | 85.84 | 85.86 | 84.19 | 85.85 | 86.07 | 88.50 | 87.73 | 88.79 | 88.55 | 90.04 | 88.95 | 90.09 | 90.16 |
| **Gm15** | 84.57 | 82.99 | 85.04 | 84.69 | 82.89 | 80.01 | 82.67 | 83.25 | 84.15 | 82.01 | 84.13 | 84.41 | 85.63 | 83.16 | 85.36 | 85.95 |
| **Gm16** | 84.62 | 84.31 | 85.04 | 84.60 | 83.79 | 82.55 | 83.56 | 83.97 | 84.28 | 83.57 | 84.29 | 84.35 | 86.04 | 84.96 | 85.83 | 86.19 |
| **Gm17** | 88.90 | 88.58 | 89.60 | 88.83 | 89.09 | 88.08 | 89.25 | 89.19 | 86.19 | 84.51 | 86.22 | 86.39 | 87.58 | 85.62 | 87.34 | 87.85 |
| **Gm18** | 83.90 | 82.90 | 84.38 | 83.95 | 83.94 | 82.34 | 83.89 | 84.14 | 84.22 | 83.05 | 84.39 | 84.33 | 85.10 | 83.50 | 84.97 | 85.31 |
| **Gm19** | 83.75 | 81.81 | 84.15 | 83.92 | 83.87 | 81.28 | 83.78 | 84.19 | 89.02 | 88.25 | 89.41 | 89.05 | 90.66 | 89.67 | 90.85 | 90.75 |
| **Gm20** | 88.34 | 88.12 | 89.06 | 88.25 | 88.56 | 87.75 | 88.82 | 88.62 | 88.68 | 88.21 | 89.12 | 88.67 | 90.17 | 89.39 | 90.38 | 90.24 |

**Table S3 Effective coverage of various regions in each sample.**

| **Regions** | **NCC-HF55** | | | | **CC-HF55** | | | | **NCC-KX2** | | | | **CC-KX2** | | | |
| --- | --- | --- | --- | --- | --- | --- | --- | --- | --- | --- | --- | --- | --- | --- | --- | --- |
|  | C | CG | CHG | CHH | C | CG | CHG | CHH | C | CG | CHG | CHH | C | CG | CHG | CHH |
| **3′-UTR** | 96.67 | 97.09 | 97.57 | 96.47 | 97.46 | 97.5 | 97.97 | 97.36 | 97.77 | 97.86 | 98.29 | 97.67 | 98.26 | 98.14 | 98.6 | 98.21 |
| **5′-UTR** | 89.84 | 94.38 | 94.46 | 88.15 | 92.11 | 95.3 | 95.48 | 90.89 | 92.56 | 95.72 | 95.85 | 91.36 | 94.47 | 96.6 | 96.85 | 93.64 |
| **CDS** | 94.05 | 93.98 | 95.27 | 93.76 | 94.46 | 94.02 | 95.4 | 94.3 | 94.66 | 94.28 | 95.56 | 94.5 | 95.59 | 94.89 | 96.26 | 95.53 |
| **Downstream** | 93.93 | 94.53 | 95.18 | 93.68 | 94.81 | 94.81 | 95.51 | 94.71 | 95.31 | 95.46 | 95.98 | 95.2 | 95.91 | 95.83 | 96.41 | 95.85 |
| **Genebody** | 94.23 | 93.55 | 95.52 | 94.05 | 94.87 | 93.69 | 95.76 | 94.82 | 95.15 | 94.07 | 95.99 | 95.09 | 95.92 | 94.6 | 96.55 | 95.93 |
| **Upstream** | 92.69 | 94 | 94.27 | 92.33 | 94 | 94.55 | 94.86 | 93.83 | 94.59 | 95.22 | 95.45 | 94.4 | 95.35 | 95.67 | 95.97 | 95.23 |

**Table S4 The primer sets used in qRT-PCR**

| **Target genes** | **Primer sequences (5′→3′)** | **Amplicon size (bp)** |
| --- | --- | --- |
| *DME* | *DME*-F TGGGACCTCCGTATCATCTATT | 165 |
|  | *DME-*R TCTGTCTTTGCTAACCTGCTTG |  |
| *DML* | *DML*-F TGGGAAGATGAACGAAATGTG | 163 |
|  | *DML*-R AACTGGAAAGATGGTCCGAGA |  |
| *ROS1* | *ROS1*-F GGTCATCGTTCCCTTTCACAT | 160 |
|  | *ROS1*-R TCGTTCTTCTTCCCACCACTT |  |
| *ACTIN* | *ACT*-F GACCTTCAACACCCCTGCT | 143 |
|  | *ACT*-R GTGGGAGTGCATAACCCTC |  |


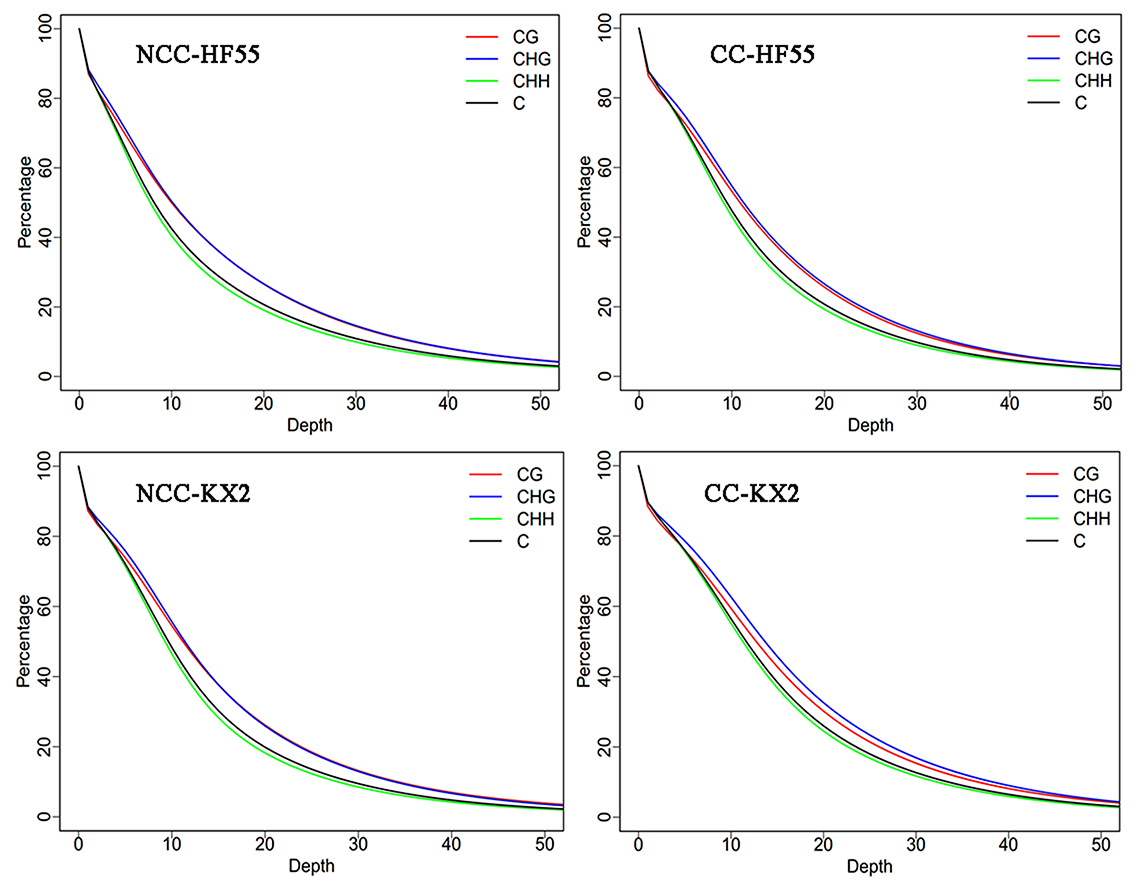


**Fig. S1 Cumulative distribution of effective sequencing depth in total cytosine and three sequence contexts.**


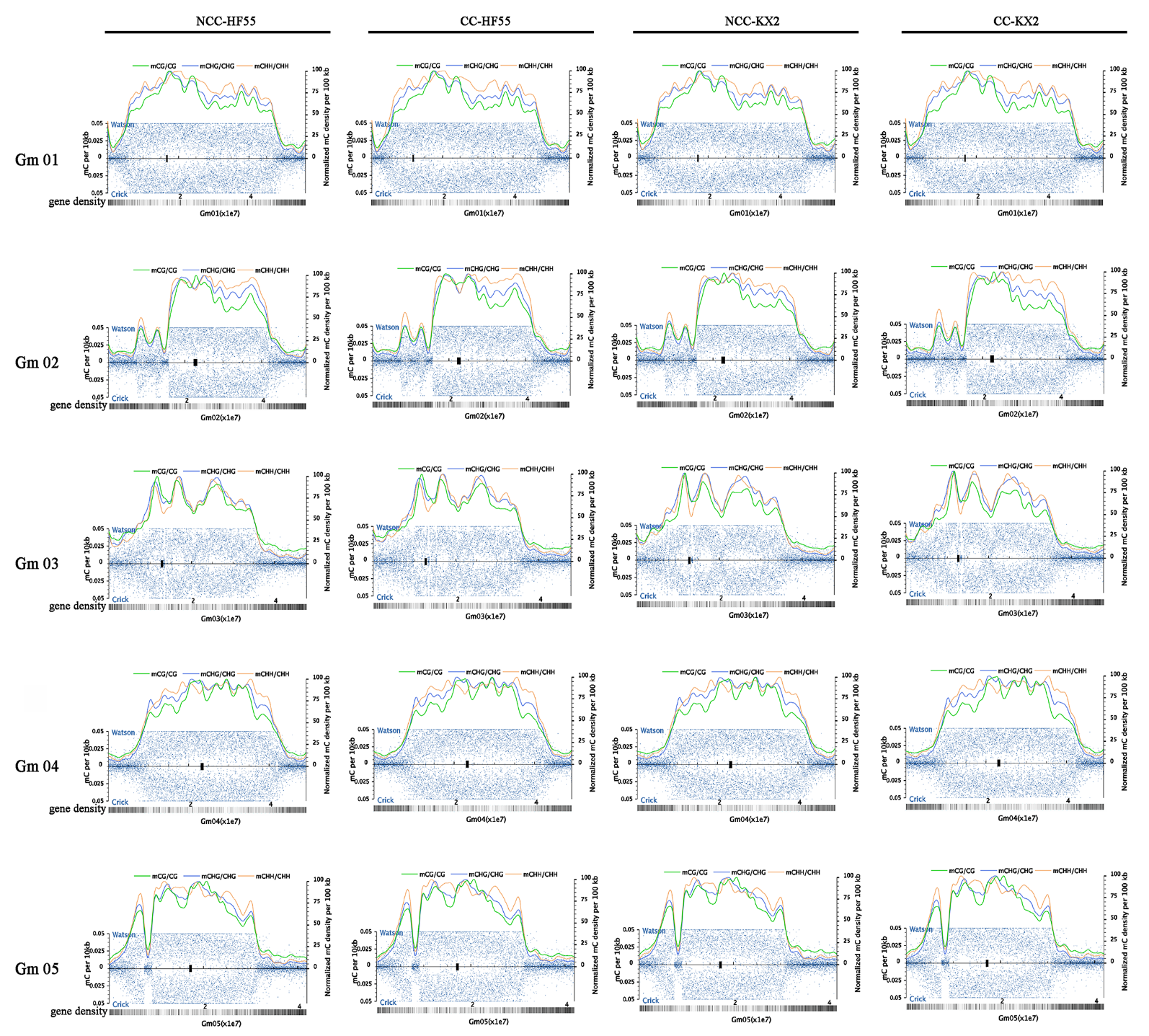


**Fig. S2-1 Methylcytosine density throughout chromosome one to five in sensitive HF55 and tolerant KX2 under different conditions.** Normalized methylated cytosine over total cytosine positions in 10-kb windows (blue dots, left axis) and normalized methylated CpG, CpHpG, and CpHpH contexts in 100-kb windows (smoothed lines, right axis).

**
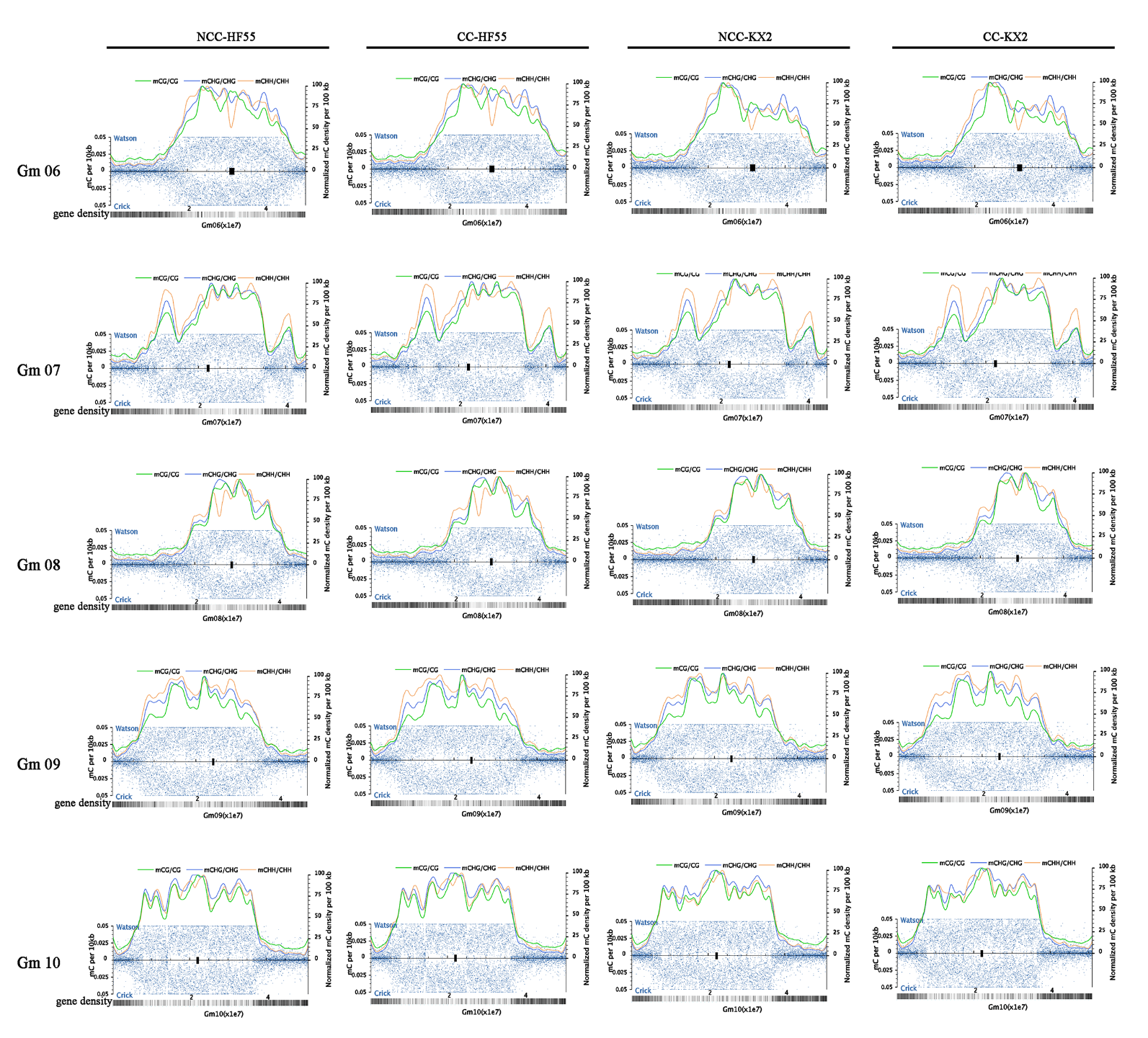
**

**Fig. S2-2 Methylcytosine density throughout chromosome six to ten in sensitive HF55 and tolerant KX2 under different conditions.** Normalized methylated cytosine over total cytosine positions in 10-kb windows (blue dots, left axis) and normalized methylated CpG, CpHpG, and CpHpH contexts in 100-kb windows (smoothed lines, right axis).

**
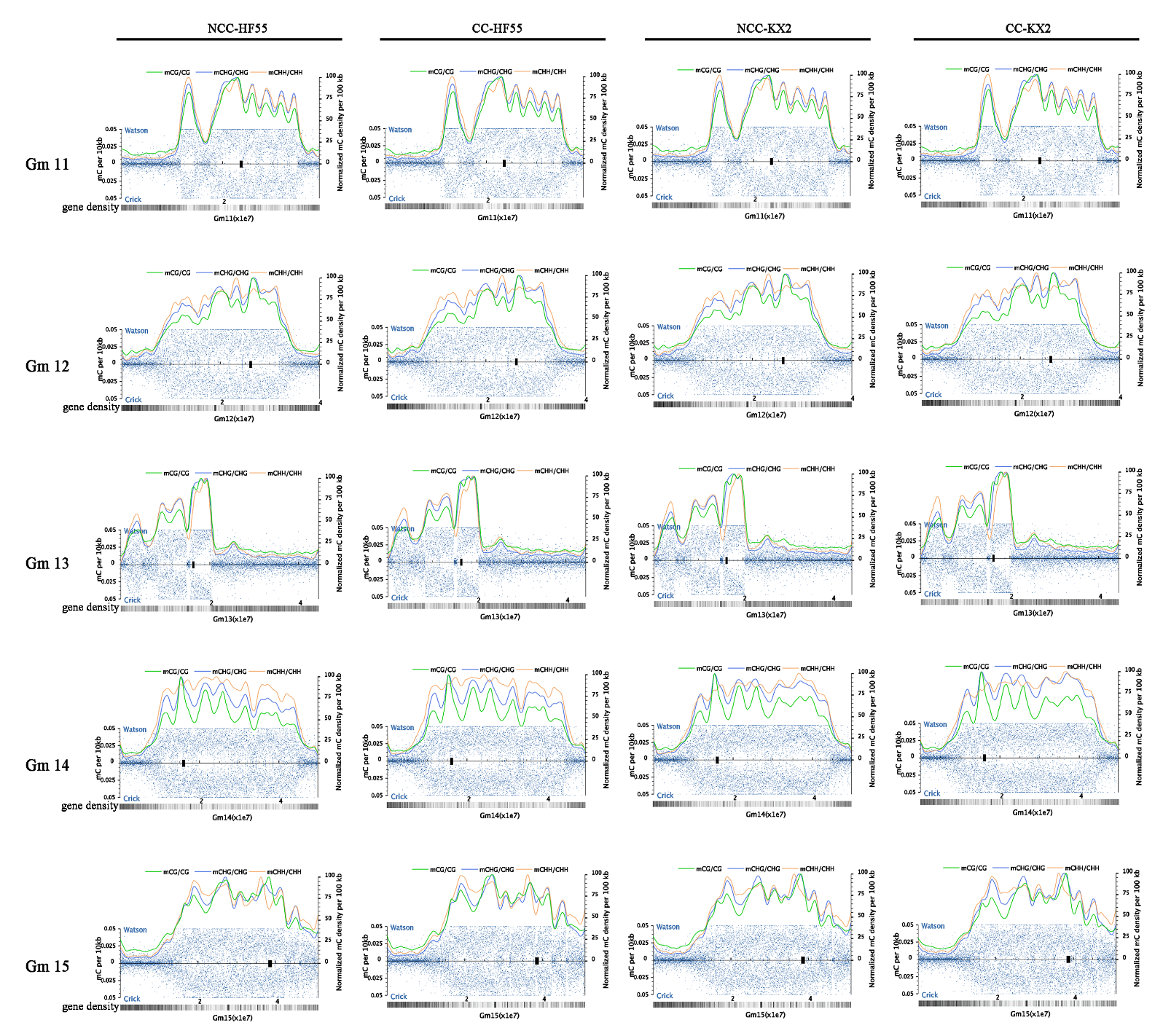
**

**Fig. S2-3 Methylcytosine density throughout chromosome eleven to fifteen in sensitive HF55 and tolerant KX2 under different conditions.** Normalized methylated cytosine over total cytosine positions in 10-kb windows (blue dots, left axis) and normalized methylated CpG, CpHpG, and CpHpH contexts in 100-kb windows (smoothed lines, right axis).

**
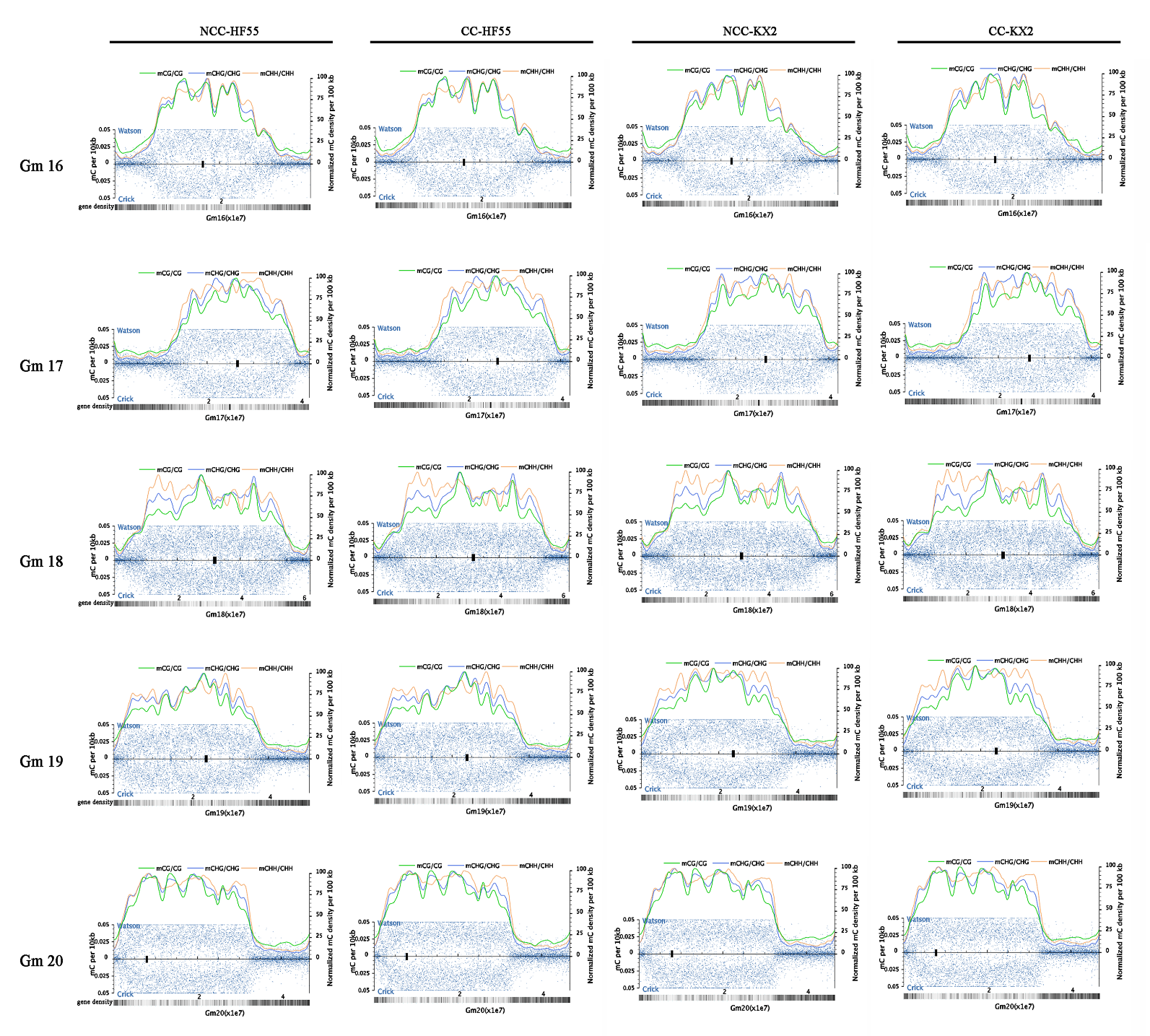
**

**Fig. S2-4 Methylcytosine density throughout chromosome sixteen to twenty in sensitive HF55 and tolerant KX2 under different conditions.** Normalized methylated cytosine over total cytosine positions in 10-kb windows (blue dots, left axis) and normalized methylated CpG, CpHpG, and CpHpH contexts in 100-kb windows (smoothed lines, right axis).
